# Identifying essential genes/reactions of the rice photorespiration by *in silico* model-based analysis

**DOI:** 10.1186/1939-8433-6-20

**Published:** 2013-08-13

**Authors:** Meiyappan Lakshmanan, Bijayalaxmi Mohanty, Dong-Yup Lee

**Affiliations:** Department of Chemical and Biomolecular Engineering, National University of Singapore, 4 Engineering Drive 4, Singapore, 117576 Singapore; Bioprocessing Technology Institute, Agency for Science, Technology and Research (A*STAR), 20 Biopolis Way, #06-01, Centros, Singapore, 138668 Singapore

**Keywords:** Rice metabolism, Photorespiration, Gene essentiality, Metabolic network model, Constraints-based flux analysis, Synthetic lethality

## Abstract

**Background:**

Photorespiration, a highly wasteful process of energy dissipation, depresses the productivity of C3 plants such as rice (*Oryza sativa*) under dry and hot conditions. Thus, it is highly required to understand the cellular physiology and relevant metabolic states under photorespiration using systems approaches, thereby devising strategies for improving rice production.

**Findings:**

*In silico* model-driven gene deletion analysis was performed on photorespiring leaf cells under ambient and stressed environmental conditions using our central metabolic network of rice cells. As a result, we identified a number of essential genes for the cell growth across various functional pathways such as photorespiratory cycle, Calvin cycle, GS-GOGAT cycle and sucrose metabolism as well as certain inter-compartmental transporters, which are mostly in good agreement with previous experiments. Synthetic lethal (SL) screening was also performed to identify the pair of non-essential genes whose simultaneous deletion become lethal, revealing the existence of more than 220 pairs of SLs on rice central metabolism.

**Conclusions:**

The gene deletion and synthetic lethal analyses highlighted the rigid nature of rice photosynthetic pathways and characterized functional interactions between central metabolic genes, respectively. The biological roles of such reported essential genes should be further explored to better understand the rice photorespiration in future.

**Electronic supplementary material:**

The online version of this article (doi:10.1186/1939-8433-6-20) contains supplementary material, which is available to authorized users.

## Findings

### *In silico* model driven analysis of rice photorespiration

Drought stress is one of the major environmental factors affecting the growth and development of rice due to high levels of photorespiration. Thus, in order to investigate how this abiotic stress affects the rice physiology via metabolic adaptations, it is essential to characterize the cellular behavior during photorespiration. The process is initiated by the oxygenase side reaction of the bifunctional ribulose-1,5-bisphosphate carboxylase/oxygenase (RuBisCO), producing equimolar amounts of 3-phosphoglycerate (3-PGA) and unwanted 2-phosphoglycolate (2-PG) for each molecule of O_2_ fixed (Jordan and Ogren 1984). It is followed by the salvage of 2-PG into 3-PGA via photorespiratory pathway, requiring significant amount of cellular energy, i.e. ATP, in C3 plants such as rice (Wingler et al. 2000). The ratio of carboxylase/oxygenase reactions (V_C_/V_O_) is three under normal conditions (Heldt and Piechulla 2011), however, this ratio can drop even below one and may reach the compensation point (V_C_/V_O_ = 0.5) at which the net CO_2_ uptake rate becomes zero under drought conditions (Heldt and Piechulla 2011). Therefore, the control of photorespiration has always been a main focus for improving rice productivity.

To date, a number of mutational studies have been performed in many C3 plants, mainly in Arabidopsis and barley, to understand the photorespiratory pathway, but identified only a handful of essential enzymes including serine-glyoxylate aminotransferase (SGAT), glycine decarboxylase (GDC), ferredoxin-dependent glutamate synthase (Fd-GOGAT) and glutamine synthase (GS) (Foyer et al. 2009) due to the limitations in mutant isolation process and the possible involvement of alternate pathways and genetic redundancy of the relevant enzymes (Reumann 2004; Timm et al. 2008). Thus, it is highly required to exploit more systematic approaches for improving our understanding of the rice photorespiration. In this regard, *in silico* metabolic modeling and analysis allow us to predict the cellular behaviour and metabolic states globally upon various environmental/genetic changes (Lewis et al. 2012). For example, *in silico* knock-out mutant studies and comparative analysis have provided the conditionally essential gene sets and corresponding functional modules under various growth conditions in *E. coli* and *S. cerevisiae* (Segre et al. 2005; Joyce et al. 2006). Similarly, in this work, we aim to elucidate the rice photorespiration under ambient and drought conditions by identifying essential genes using our recently reconstructed rice metabolic/regulatory network model which was validated with cell culture experiments (Lakshmanan et al. 2013).

### Identification of essential genes in rice photorespiration

**Figure 1:**
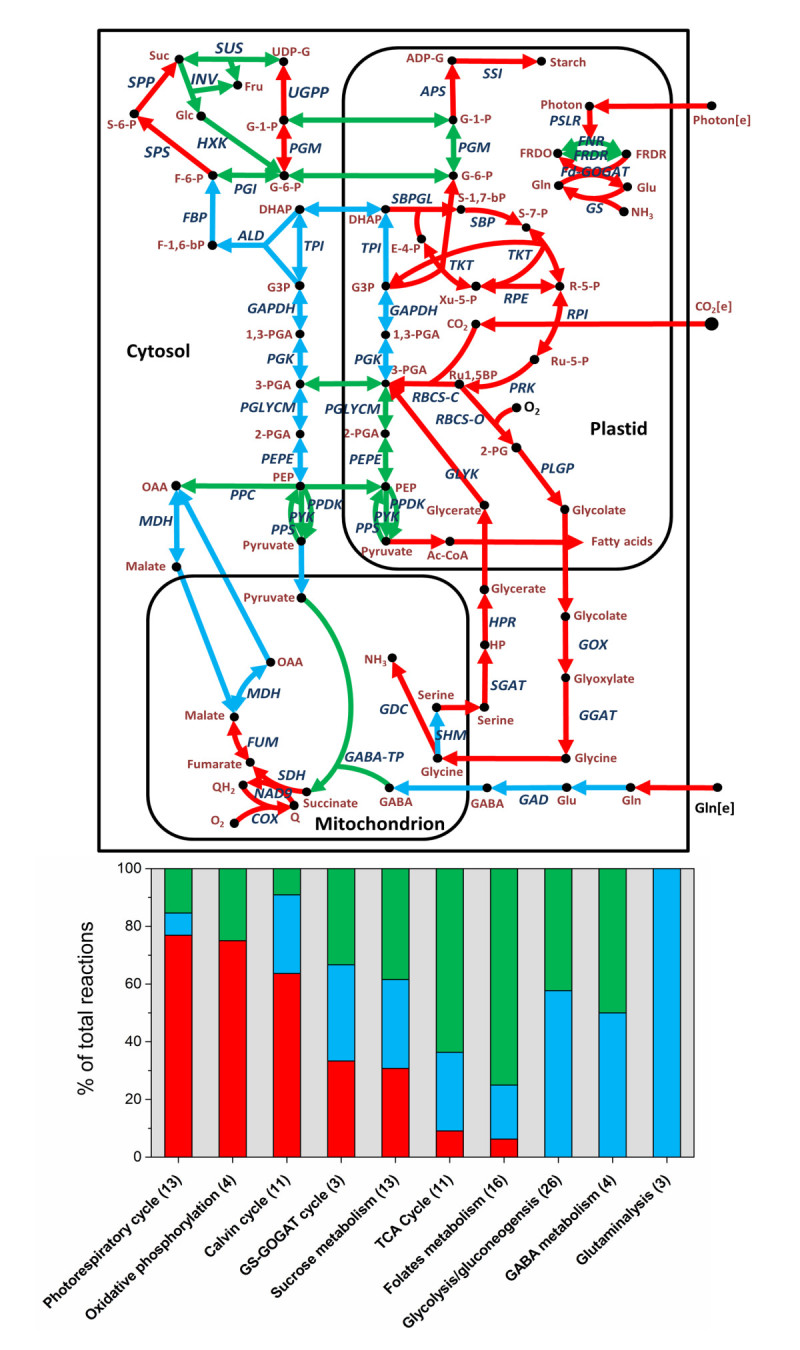
**Distribution of the essential genes across rice central metabolism in photorespiring rice leaf cells under normal (V**_**C**_**/V**_**O**_ **= 3) condition.** The genes are classified as essential (green), sub essential (blue) and non-essential (green) upon the gene deletion.

Both normal (V_C_/V_O_ = 3) and stressed (V_C_/V_O_ = 1) conditions during the rice photorespiration were simulated to evaluate the gene essentiality for cell growth by resorting to constraints-based flux analysis (see Method in Additional file 1). The results revealed about 60% of the reactions in the model were non-essential under both conditions while 25% were completely essential and distributed across various pathways of rice central metabolism as illustrated in Figure 1. Most of the essential genes were identified in photosynthetic pathways such as photorespiratory cycle (10 genes) and Calvin cycle (7 genes), indicating the rigidity of CO_2_ fixing mechanism in plants. Generally, these observations are in good agreement with the existing experimental evidences available on other plants such as Arabidopsis, pea, barley and maize (Table 1). The first enzyme of the photorespiratory pathway, phosphoglycolate phosphatase (PGLP), metabolizes the toxic 2-PG which may accumulate as a result of ribulose-1,5-bisphosphate (RuBP) oxygenase activity. If the 2-PG is not scavenged, it can inhibit the key glycolytic enzyme, triose phosphate isomerase (TPI), thereby disrupting photosynthesis even under ambient air (Somerville and Ogren 1979). Likewise, other photorespiratory enzymes such as glycolate oxidase (GOX) and SGAT are also essential for cell growth under both normal and stressed conditions by degrading the toxic metabolites, glycolate and glyoxylate, respectively (Zelitch et al. 2009; Wingler et al. 2000). Interestingly, serine hydroxymethyltransferase (mitochondrial) (SHM1: EC. 2.1.2.1), on the other hand, was found to be essential only under dry and hot conditions, which is highly consistent with the experiments by Voll et al. (2006), who reported the conditional viability of the SHM1 mutant of *Arabidopsis thaliana*. In order to further verify the results in rice, such predicted genes on Calvin cycle, photorespiratory pathway and GS-GOGAT cycle were compared with the essential genes of Arabidopsis and maize (Wang et al. 2012). Again, most of essential enzymes are common across all three plants, except PGLP with supporting experiments for our prediction (Somerville and Ogren 1979).

**Table 1:** **Comparison of essential genes/reactions in rice, Arabidopsis and maize during photorespiration**

Enzyme	EC number	Pathway	Rice (C3)	Arabidopsis (C3)	Maize (C4)	References
			(This study)	Wang et al. (2012)		
RuBisCO	4.1.1.39	Calvin cycle	√	√	√	Sicher and Bunce (1997)
PRK	2.7.1.19	Calvin cycle	√	√	√	Moll and Levine (1970)
RPE	5.3.1.6	Calvin cycle	√	√	√	
RPI	5.1.3.1	Calvin cycle	√	√	√	
TKT	2.2.1.1	Calvin cycle	√	√	√	
SBPase	3.1.3.37	Calvin cycle	√	NA	NA	Liu et al. (2012)
PLGP	3.1.3.18	Photorespiratory cycle	√	X	X	Somerville and Ogren (1979)
SHM	2.1.2.1	Photorespiratory cycle	√*	NA	NA	Voll et al. (2006)
GLYK	2.7.1.31	Photorespiratory cycle	√	NA	NA	Boldt et al. (2005)
GDC		Photorespiratory cycle	√	NA	NA	Wingler et al. (1997)
Catalase	1.11.1.6	Photorespiratory cycle	√	NA	NA	
GAL	6.3.1.2	Photorespiratory cycle	√	NA	NA	
SGAT	2.6.1.45	Photorespiratory cycle	√	NA	NA	Wingler et al. (1999)
GOX	1.1.3.15	Photorespiratory cycle	√	√	√	Zelitch et al. (2009)
GS	4.2.1.2	GS-GOGAT cycle	√	NA	NA	Blackwell et al. (1987)
Fd-GOGAT	3.1.3.24	GS-GOGAT cycle	√	NA	NA	Somerville and Ogren (1986)
GLBE	2.4.1.18	Starch biosynthesis	√	√	√	
PPC	4.1.1.31		X	X	√	

√ – essential gene; X – non-essential gene; NA– essentiality not reported/investigated; * – essential only under drought conditions.

In addition to essential metabolic genes, we also analysed the dispensability of inter-compartmental metabolite transporters since the mechanism of photorespiration is quite intricate, involving three major organelles, chloroplast, mitochondrion and peroxisome. In this regard, we identified a number of essential inter-compartmental transporters including mitochondrial and plastidic malate/fumarate/succinate redox shuttles in both conditions. Malate transporters play an essential role in transmitting the excess redox cofactors from plastid to mitochondria for their eventual utilization in oxidative phosphorylation while plastidic glycolate/glycerate transporter (PLGG) was reported as the core photorespiratory transporter (Pick et al. 2013). Additionally, a few unique mitochondrial transporters such as serine translocator, alpha-ketoglutarate/malate and glutamate/malate redox shuttles were identified to be essential only under stressed conditions, emphasizing their crucial roles in transporting the high fluxes of photorespiratory intermediates such as glycolate, glycerate, glutamate, oxoglutarate, glycine, and serine (Reumann and Weber 2006) (see Additional file 2 for the entire list of essential transporters).

From our analysis, it is evident that most of the photorespiratory enzymes including PGLP, GOX and SGAT are required for degrading the toxic metabolites and synthesizing signaling compounds such as H_2_O_2_ and glutathione (Wingler et al. 2000). Therefore, more carbohydrates and energy production via Calvin cycle toward the enhanced crop productivity can be achieved by increasing CO_2_ concentration around RuBisCO rather than blocking carbon fluxes through photorespiratory pathway.

### Synthetic lethality screening of non-essential gene pairs in rice photorespiration

Besides identifying essential genes/reactions, we also newly screened the synthetic lethal (SL) gene pairs of rice central metabolism during photorespiration under normal and stressed conditions to better characterize the functional interactions between the non-essential genes (see Methods in Additional file 1). Note that SLs are pair of non-essential genes whose simultaneous removal can lead to zero growth (Suthers et al. 2009). Such lethality arises due to several reasons including interchangeable gene products with respect to an essential function (isozymes/isoforms), their existence in the same essential pathway or sharing of complementary essential function(s) (Suthers et al. 2009). Here, it should be noted that we excluded the inter-compartmental transporters during SL screening since the deletion of most of the transporters coupled with metabolic genes resulted in no growth. A total of 226 and 229 SLs were identified in the normal and stressed conditions, respectively. Interestingly, of the total 226 SLs the ferredoxin-NADP^+^ reductase (FNR) in GS-GOGAT cycle, and the mitochondrial ATP synthase (ATPS) in oxidative phosphorylation were paired with 83 and 82 other genes of rice central metabolism, respectively. FNR is involved in reassimilating the ammonia released during photorespiration via Fd-GOGAT and maintaining the redox balance of plastids (Foyer et al. 2009) whereas ATPS is utilized to generate necessary energy for the cell growth via mitochondrial respiration (Lakshmanan et al. 2013). Several SLs also contained the isoforms of same enzymes across different compartments. Such examples include the cytosolic and plastidic isoforms of enolase, phosphoglycerate kinase, glyceraldehyde-3-phosphate dehydrogenase and triosephosphate isomerase, and the cytosolic and mitochondrial isoforms of malate dehydrogenase (see Additional file 2 for the entire list of SLs).

### Concluding remarks

In the present study, we reported the essential genes and synthetic lethal gene pairs of rice central metabolism during photorespiration using *in silico* model-driven analysis. Our model simulations have unraveled several new essential genes of the photorespiratory metabolism, in addition to those which are reported earlier. However, it should be noted that gene essentiality results are condition-specific and sensitive to the model completeness. Therefore, the list of essential genes presented in the current study should be further confirmed with enhanced model predictability and subsequent experimental validations.

## Electronic supplementary material

Additional file 1: Details of the methods used in the study. (PDF 297 KB)

Additional file 2: List of essential inter-compartmental transporters and list of synthetic lethal gene pairs of rice central metabolism under normal and stressed photorespiration. (PDF 128 KB)

Below are the links to the authors’ original submitted files for images.

Authors’ original file for figure 1

## Abbreviations

2-PG: 2-phosphoglycolate
3-PGA: 3-phosphoglycerate
ATPS: ATP synthase
CAT: Catalase
Fd-GOGAT: Ferredoxin-dependent glutamate synthase
FNR: Ferredoxin-NADP + reductase (plastidic)
GAL: Glutamate - ammonia ligase
GDC: Glycine decarboxylase
GLBE: 1,4-alpha-glucan branching enzyme
GLYK: Glycerate kinase
GOX: Glycolate oxidase
GS: Glutamine synthase
HPR: Hydroxypyruvate reductase
PGLP: Phosphoglycolate phosphatase
PLGG: Plastidic glycolate/glycerate translocator
PRK: Phosphoribulokinase
RPE: Ribulose phosphate 3-epimerase
RPI: Ribulose phosphate 3-isomerase
RuBisCO: D-ribulose-1,5-bisphosphate carboxylase/oxygenase
RuBP: D-ribulose-1,5-bisphosphate
SBPase: Sedoheptulose-bisphosphatase
SGAT: Serine-glyoxylate aminotransferase
SHM: Serine hydroxymethyltransferase
SL: Synthetic lethal
SPP: Sucrose-phosphatase
TKT: Transketolase
TPI: Triose phosphate isomerase.
