# Supplementary material for: Identifying essential genes/reactions of the rice photorespiration by in silico model-based analysis
Source: Rice (N Y). 2013 Aug 13;6:20. doi: 10.1186/1939-8433-6-20 (PMC4883722; doi:10.1186/1939-8433-6-20)
Supplement: Supplementary file 1 — Additional file 1: Details of the methods used in the study. (PDF 297 KB) [file 12284_2013_55_MOESM1_ESM.pdf]

# Additional file 1

## Identifying essential genes of the rice photorespiration by *in silico* model-based analysis

Meiyappan Lakshmanan, Bijayalaxmi Mohanty, Dong-Yup Lee

### METHOD

#### Constraints-based flux analysis

In this study, we utilized constraints-based flux analysis to identify the essential genes/reactions in the rice metabolism under varying environmental conditions by manipulating the constraints. The biomass equation was maximized to obtain the optimal solution of the metabolic network as detailed elsewhere (Oberhardt et al., 2009). Mathematically, the problem specific to our study, i.e. maximization of biomass subjected to stoichiometric and flux capacity constraints can be represented as follows:

$$\begin{aligned} &\max v_{biomass} \\ &\text{s.t.} \quad \sum_j S_{ij} v_j = 0 \quad \forall \text{ metabolite } i \\ &\qquad\; v_j^{\max} \le v_j \le v_j^{\min} \quad \forall \text{ reaction } j \end{aligned}$$

where $S_{ij}$ refers to the stoichiometric coefficient of metabolite *i* involved in reaction *j*, $v_j$ denotes to the flux or specific rate of metabolic reaction *j*, $v_j^{\min}$ and $v_j^{\max}$ represent the lower and upper limits on the flux of reaction *j*, respectively; and *Z* corresponds to the cellular objective as a linear function of all the metabolic reactions where the relative weights are determined by the coefficient $c_j$.

In order to simulate the reaction deletions under photorespiration, the leaf cell growth was simulated by maximizing the leaf biomass while constraining flux through the corresponding reaction to be zero under a defined the photon uptake at 100 mmol $g^{-1}$ DCW $day^{-1}$. In addition, the ratio of flux through *RuBisCO* was set with a value of either three or one representing the normal and stressed conditions (Weber, 2007). In this study, all simulations were implemented by General Algebraic Modeling System (GAMS) Integrated Development Environment (IDE) version 23.9.

**References**

Oberhardt MA, Chavali AK, Papin JA (2009) Flux balance analysis: interrogating genome-scale metabolic networks. Methods in Molecular Biology 500: 61-80

Weber APM (2007) Synthesis, Export and Partitioning of the End Products of Photosynthesis. In RR Wise, JK Hoober, eds, The Structure and Function of Plastids. Springer, pp 273-292

# MATERIAL

## Rice central metabolic model

| Reaction ID | Reaction Name | Reaction Equation | Pathway | EC No. |
|---|---|---|---|---|
| Straw_Biomass | Straw Biomass reaction | 0.0321 Ala[c] + 0.0123 Arg[c] + 0.0417 Asp[c] + 0.0033 Cys[c] + 0.0385 Glu[c] + 0.0282 Gly[c] + 0.057 His[c] + 0.0140 Ile[c] + 0.0234 Leu[c] + 0.0153 Lys[c] + 0.0076 Met[c] + 0.0133 Phe[c] + 0.0225 Pro[c] + 0.0219 Ser[c] + 0.0192 Thr[c] + 0.0016 Trp[c] + 0.0077 Tyr[c] + 0.0225 Val[c] + 0.0546 a-Glc[c] + 0.1049 Sucrose[c] + 0.0643 Fruct[c] + 0.3249 Starch[p] + 2.0789 cellulose[c] + 0.1256 UDP-L-arab[c] + 0.5720 UDP-Xyl[c] + 0.0229 UDP-Gal[c] + 0.4018 UDP-Glc[c] + 0.5033 UDP-Glucur[c] + 0.2065 Coum-Alc[c] + 0.1721 Conalc[c] + 0.1475 SinapAlc[c] + 0.01625 TAG[c] + 41.75 ATP[c] -> 41.75 ADP[c] + 41.75 pi[c] + 1.6256 UDP[c] + 1.6256 H+[c] | Biomass reactions | |
| Ex-CO2 | Carbon dioxide exchange | CO2[e] <-> CO2[c] | Exchange Reactions | |
| Ex-H2O | Water exchange | H2O[e] <-> H2O[c] | Exchange Reactions | |
| Ex-photon | Photon exchange | photon[e] -> photon[p] | Exchange Reactions | |
| Ex-H+ | Proton exchange | H+[e] <-> H+[c] | Exchange Reactions | |
| Ex-O2 | Oxygen exchange | O2[e] <-> O2[c] | Exchange Reactions | |
| Ex-pp | Phosphate exchange | pi[e] <-> pi[c] | Exchange Reactions | |
| Ex-Sulfate | Sulfate exchange | sulfate[e] <-> sulfate[c] | Exchange Reactions | |
| Ex-Asparagine | Asparagine exchange | Asn[e] <-> Asn[c] | Exchange Reactions | |
| Ex-Glutamine | Glutamine exchange | Gln[e] <-> Gln[c] | Exchange Reactions | |
| Ex-ethanol | Ethanol exchange | Ethanol[c] <-> Ethanol[e] | Exchange Reactions | |
| Ex-Lactate | Lactate exchange | Lactate[c] <-> Lactate[e] | Exchange Reactions | |
| Ex-Alanine | Alanine exchange | Ala[c] <-> Ala[e] | Exchange Reactions | |
| Ex-Acetate | Acetate exchange | Acetate[c] <-> Acetate[e] | Exchange Reactions | |
| Ex-Succinate | Succinate exchange | Succ[c] <-> Succ[e] | Exchange Reactions | |
| IPP[c] | Inorganic pyrophosphatase | H2O[c] + ppi[c] -> 2 pi[c] + H+[c] | Sucrose metabolism | 3.6.1.1 |
| IPP[m] | Inorganic pyrophosphatase | H2O[m] + ppi[m] -> 2 pi[m] + H+[m] | Sucrose metabolism | 3.6.1.1 |

| | | | | |
|---|---|---|---|---|
| IPP[p] | Inorganic pyrophosphatase | H2O[p] + ppi[p] -> 2 pi[p] + H+[p] | Sucrose metabolism | 3.6.1.1 |
| ALS1[p] | Acetolactate synthase | 2 Pyr[p] + H+[p] -> 2Acelac[p] + CO2[p] | Valine biosynthesis | 2.2.1.6 |
| CAT[c] | Catalase | 2 H2O2[c] -> 2 H2O[c] + O2[c] | Photorespiration | 1.11.1.6 |
| GLU1[p] | Glutamate synthase (ferredoxin) | 2 redferr[p] + aKG[p] + Gln[p] + 2 H+[p] -> 2 oxiferr[p] + 2 Glu[p] | GS-GOGAT Cycle | 1.4.7.1 |
| RBCS-C[p] | Ribulose-bisphosphate carboxylase | RuBP[p] + CO2[p] + H2O[p] -> 2 3PG[p] + 2 H+[p] | Calvin cycle | 4.1.1.39 |
| COX[m] | Cytochrome c oxidase (complex IV) | 4 Cyto-Red[m] + O2[m] + 6 H+[m] -> 4 Cyto-Oxi[m] + 2 H2O[m] + 6 H+[c] | Oxidative phosphorylation | 1.9.3.1 |
| ATPPH[c] | ATP phosphohydrolase | ATP[c] + H2O[c] -> ADP[c] + pi[c] + H+[c] | Purine metabolism | 3.6.1.3 |
| ATPS[m] | ATP synthase (complex V) | ADP[m] + pi[m] + 3 H+[c] -> ATP[m] + H2O[m] + 2 H+[m] | Oxidative phosphorylation | 3.6.3.14 |
| GRC2[p] | Glutathione reductase (NADPH) | GludiS[p] + NADPH[p] + H+[p] -> 2 Gluta[p] + NADP+[p] | Sulfate assimilation | 1.8.1.7 |
| ADNK[c] | Adenylate kinase | AMP[c] + ATP[c] -> 2 ADP[c] | Purine metabolism | 2.7.4.3 |
| ADNK[p] | Adenylate kinase | AMP[p] + ATP[p] -> 2 ADP[p] | Purine metabolism | 2.7.4.3 |
| NDKR1[c] | nucleoside-diphosphate kinase | UDP[c] + ATP[c] <-> UTP[c] + ADP[c] | Pyrimidine metabolism | 2.7.4.6 |
| NDKR1[p] | nucleoside-diphosphate kinase | UDP[p] + ATP[p] <-> UTP[p] + ADP[p] | Pyrimidine metabolism | 2.7.4.6 |
| ATPT[p] | ATP:UMP phosphotransferase | ATP[p] + UMP[p] -> ADP[p] + UDP[p] | Pyrimidine metabolism | 2.7.4.4 |
| SAM[c] | Methionine adenosyltransferase | ATP[c] + Met[c] + H2O[c] -> pi[c] + ppi[c] + S-Ade-L-meth[c] | Methionine biosynthesis | 2.5.1.6 |
| AMPP[c] | Adenosine 5'-monophosphate phosphohydrolase | AMP[c] + H2O[c] -> Adenosine[c] + pi[c] | Purine metabolism | 3.1.3.5 |
| ADK[c] | Adenosine kinase | Adenosine[c] + ATP[c] -> AMP[c] + ADP[c] | Purine metabolism | 2.7.1.20 |
| SAHH[c] | Adenosylhomocysteinase | S-Ade-L-H[c] + H2O[c] -> HomoCys[c] + Adenosine[c] | Methionine biosynthesis | 3.3.1.1 |
| PPS[c] | Pyruvate,water dikinase | H2O[c] + Pyr[c] + ATP[c] -> pi[c] + PEP[c] + AMP[c] | Glycolysis/Gluconeogensis | 2.7.9.2 |
| PPS[p] | Pyruvate,water dikinase | H2O[p] + Pyr[p] + ATP[p] -> pi[p] + PEP[p] + AMP[p] | Glycolysis/Gluconeogensis | 2.7.9.2 |
| PYK[c] | Pyruvate kinase | ADP[c] + PEP[c] + H+[c] -> ATP[c] + Pyr[c] | Glycolysis/Gluconeogensis | 2.7.1.40 |

| | | | | |
|---|---|---|---|---|
| PYK[p] | Pyruvate kinase | ADP[p] + PEP[p] + H+[p] -> ATP[p] + Pyr[p] | Glycolysis/Gluconeogensis | 2.7.1.40 |
| PPDK1[p] | Pyruvate phosphate dikinase | ppi[p] + PEP[p] + AMP[p] <-> ATP[p] + Pyr[p] + pi[p] | Glycolysis/Gluconeogensis | 2.7.9.1 |
| PPDK2[c] | Pyruvate phosphate dikinase | ppi[c] + PEP[c] + AMP[c] <-> ATP[c] + Pyr[c] + pi[c] | Glycolysis/Gluconeogensis | 2.7.9.1 |
| PFL[c] | Formate C-acetyltransferase | CoA[c] + Pyr[c] -> Formate[c] + Ace-CoA[c] | Fermentation | 2.3.1.54 |
| ME6[p] | Malate dehydrogenase (oxaloacetate decarboxylating) (NADP+) | NADP+[p] + Malate[p] -> NADPH[p] + CO2[p] + Pyr[p] | Photorespiration | 1.1.1.40 |
| PDC[c] | pyruvate decarboxylase | Pyr[c] + H+[c] -> Acetald[c] + CO2[c] | Fermentation | 4.1.1.1 |
| ACDH[c] | Acetaldehyde dehydrogenase (acetylating) | NAD+[c] + CoA[c] + Acetald[c] <-> NADH[c] + Ace-CoA[c] + H+[c] | Fermentation | 1.2.1.10 |
| ACS[c] | acetate--CoA ligase | CoA[c] + Acetate[c] + ATP[c] -> Ace-CoA[c] + ppi[c] + AMP[c] | Fermentation | 6.2.1.1 |
| ACS[m] | acetate--CoA ligase | CoA[m] + Acetate[m] + ATP[m] -> Ace-CoA[m] + ppi[m] + AMP[m] | Fermentation | 6.2.1.1 |
| ACS[p] | acetate--CoA ligase | CoA[p] + Acetate[p] + ATP[p] -> Ace-CoA[p] + ppi[p] + AMP[p] | Fermentation | 6.2.1.1 |
| P5CS1[c] | Glutamate 5-kinase | Glu[c] + ATP[c] -> L-Glut-5P[c] + ADP[c] | Proline metabolism | 2.7.2.11 |
| P5CS1[p] | Glutamate 5-kinase | Glu[p] + ATP[p] -> L-Glut-5P[p] + ADP[p] | Proline metabolism | 2.7.2.11 |
| GDH1[m] | Glutamate dehydrogenase (NADP+) | ammonia[m] + aKG[m] + NADPH[m] + H+[m] -> Glu[m] + H2O[m] + NADP+[m] | Glutamine/Glutamate metabolism | 1.4.1.4 |
| GDH2[m] | Glutamate dehydrogenase (NAD+) | Glu[m] + H2O[m] + NAD+[m] -> ammonia[m] + aKG[m] + NADH[m] + H+[m] | Glutamine/Glutamate metabolism | 1.4.1.2 |
| GLN1[c] | Glutamate--ammonia ligase | ammonia[c] + Glu[c] + ATP[c] -> Gln[c] + ADP[c] + pi[c] | Glutamine/Glutamate metabolism | 6.3.1.2 |
| GLN2[p] | Glutamate--ammonia ligase | ammonia[p] + Glu[p] + ATP[p] -> Gln[p] + ADP[p] + pi[p] | Photorespiration | 6.3.1.2 |
| ALAAT[c] | Alanine aminotransferase | aKG[c] + Ala[c] <-> Glu[c] + Pyr[c] | Alanine biosynthesis | 2.6.1.2 |
| GAT[c] | Amino-acid N-acetyltransferase | Glu[c] + Ace-CoA[c] -> N-Ace-L-glutt[c] + CoA[c] | Arginine biosynthesis | 2.3.1.1 |
| GAD[c] | Glutamate decarboxylase | Glu[c] + H+[c] -> CO2[c] + GABA[c] | GABA metabolism | 4.1.1.15 |

| | | | | |
|---|---|---|---|---|
| IDP[c] | Isocitrate dehydrogenase (NADP+) | Isocit[c] + NADP+[c] -> NADPH[c] + aKG[c] + CO2[c] | TCA Cycle | 1.1.1.42 |
| IDP[m] | Isocitrate dehydrogenase (NADP+) | Isocit[m] + NADP+[m] -> NADPH[m] + aKG[m] + CO2[m] | TCA Cycle | 1.1.1.42 |
| GPX[p] | glutathione peroxidase | H2O2[p] + 2 Gluta[p] -> GludiS[p] + 2 H2O[p] | Sulfate assimilation | 1.11.1.9 |
| UGD[c] | UDP-glucose 6-dehydrogenase | UDP-Glc[c] + H2O[c] + 2 NAD+[c] -> UDP-Glucur[c] + 2 NADH[c] + 3 H+[c] | Cell wall metabolism | 1.1.1.22 |
| UGP[c] | UTP--glucose-1-phosphate uridylyltransferase | a-Glc1P[c] + UTP[c] <-> UDP-Glc[c] + ppi[c] | Sucrose metabolism | 2.7.7.9 |
| GALE[c] | UDP-glucose 4-epimerase | UDP-Glc[c] <-> UDP-Gal[c] | Cell wall metabolism | 5.1.3.2 |
| NDKR2[c] | nucleoside-diphosphate kinase | GDP[c] + ATP[c] <-> GTP[c] + ADP[c] | Purine metabolism | 2.7.4.6 |
| NDKR2[p] | nucleoside-diphosphate kinase | GDP[p] + ATP[p] <-> GTP[p] + ADP[p] | Purine metabolism | 2.7.4.6 |
| PCKA[c] | Phosphoenolpyruvate carboxykinase (ATP) | OAA[c] + ATP[c] -> CO2[c] + PEP[c] + ADP[c] | Glycolysis/Gluconeogensis | 4.1.1.49 |
| MDH[c] | Malate dehydrogenase | Malate[c] + NAD+[c] + H+[c] <-> OAA[c] + NADH[c] | Glycolysis/Gluconeogensis | 1.1.1.37 |
| MDH[m] | Malate dehydrogenase | Malate[m] + NAD+[m] + H+[m] <-> OAA[m] + NADH[m] | TCA Cycle | 1.1.1.37 |
| PPC[c] | Phosphoenolpyruvate carboxylase | pi[c] + OAA[c] <-> PEP[c] + HCO3[c] | Glycolysis/Gluconeogensis | 4.1.1.31 |
| CSY[m] | Citrate synthase | OAA[m] + Ace-CoA[m] + H2O[m] -> Citrate[m] + CoA[m] + H+[m] | TCA Cycle | 2.3.3.1 |
| ASP1[c] | Aspartate aminotransferase | Asp[c] + aKG[c] <-> Glu[c] + OAA[c] | Aspartate biosynthesis | 2.6.1.1 |
| ASP1[m] | Aspartate aminotransferase | Asp[m] + aKG[m] <-> Glu[m] + OAA[m] | Aspartate biosynthesis | 2.6.1.1 |
| ASP1[p] | Aspartate aminotransferase | Asp[p] + aKG[p] <-> Glu[p] + OAA[p] | Aspartate biosynthesis | 2.6.1.1 |
| GGAT[c] | Glycine aminotransferase | Glu[c] + Glyoxylate[c] -> aKG[c] + Gly[c] | Photorespiration | 2.6.1.4 |
| SUCLG[m] | Succinate--CoA ligase (ADP-forming) | Succ[m] + CoA[m] + ATP[m] <-> Suc-CoA[m] + ADP[m] + pi[m] | TCA Cycle | 6.2.1.5 |
| LYSA[p] | Diaminopimelate decarboxylase | mPime[p] + H+[p] -> CO2[p] + Lys[p] | Lysine biosynthesis | 4.1.1.20 |
| MLS[p] | Malate synthase | Ace-CoA[p] + H2O[p] + Glyoxylate[p] -> Malate[p] + CoA[p] | Photorespiration | 2.3.3.9 |

| | | | | |
|---|---|---|---|---|
| | | + H+[c] | | |
| GOX[c] | glycolate oxidase | Glycolate[c] + O2[c] -> Glyoxylate[c] + H2O2[c] | Photorespiration | 1.1.3.15 |
| ICL[c] | Isocitrate lyase | Isocit[c] -> Glyoxylate[c] + Succ[c] | Glyoxylate Cycle | 4.1.3.1 |
| AK[p] | Aspartate kinase | Asp[p] + ATP[p] -> Asp4P[p] + ADP[p] | Threonine biosynthesis | 2.7.2.4 |
| ASN1[p] | Aspartate--ammonia ligase | ammonia[p] + Asp[p] + ATP[p] -> Asn[p] + ppi[p] + AMP[p] | Asparagine biosynthesis | 6.3.1.1 |
| ASPG[c] | Asparaginase | Asn[c] + H2O[c] -> ammonia[c] + Asp[c] + H+[c] | Asparagine biosynthesis | 3.5.1.1 |
| FDH[c] | Formate dehydrogenase | Formate[c] + NAD+[c] -> CO2[c] + NADH[c] | Folates metabolism | 1.2.1.2 |
| SADN[p] | Sulfate adenylyltransferase | sulfate[p] + ATP[p] + H+[p] -> Ade-5P[p] + ppi[p] | Sulfate assimilation | 2.7.7.4 |
| ARGD[c] | Arginine deiminase | H2O[c] + Arg[c] -> ammonia[c] + citrulline[c] | Proline metabolism | 3.5.3.6 |
| CPA[c] | carbamoyl-phosphate synthetase (glutamine-hydrolysing) | 2 ATP[c] + Gln[c] + HCO3[c] + H2O[c] -> Glu[c] + 2 ADP[c] + pi[c] + CarmteP[c] | Arginine biosynthesis | 6.3.5.5 |
| ASN2[p] | asparagine synthetase (glutamine-hydrolysing) | Gln[p] + Asp[p] + ATP[p] + H2O[p] -> Glu[p] + Asn[p] + ppi[p] + AMP[p] + H+[p] | Asparagine biosynthesis | 6.3.5.4 |
| PSP[p] | Phosphoserine phosphatase | 3PhosSer[p] + H2O[p] -> Ser[p] + pi[p] | Serine biosynthesis | 3.1.3.3 |
| SAT[p] | Serine O-acetyltransferase | Ser[p] + Ace-CoA[p] -> O-Ace-L-ser[p] + CoA[p] | Cysteine biosynthesis | 2.3.1.30 |
| SGAT[c] | Serine--glyoxylate aminotransferase | Glyoxylate[c] + Ser[c] -> Hydpyr[c] + Gly[c] | Photorespiration | 2.6.1.45 |
| MMT[c] | Methionine S-methyltransferase | Met[c] + S-Ade-L-meth[c] -> S-Ade-L-H[c] + SMLM[c] | Methionine biosynthesis | 2.1.1.12 |
| HMT1[c] | Homocysteine S-methyltransferase | HomoCys[c] + S-Ade-L-meth[c] -> S-Ade-L-H[c] + Met[c] | Methionine biosynthesis | 2.1.1.10 |
| HMT2[c] | Homocysteine S-methyltransferase | SMLM[c] + HomoCys[c] -> 2 Met[c] | Methionine biosynthesis | 2.1.1.10 |
| ENO1[c] | Phosphopyruvate hydratase | 2PG[c] <-> PEP[c] + H2O[c] | Glycolysis/Gluconeogensis | 4.2.1.11 |
| ENO1[p] | Phosphopyruvate hydratase | 2PG[p] <-> PEP[p] + H2O[p] | Glycolysis/Gluconeogensis | 4.2.1.11 |
| CTL[c] | Citrullinase | citrulline[c] + H2O[c] + H+[c] -> Orn[c] + CO2[c] + ammonia[c] | Proline metabolism | 3.5.1.20 |

| | | | | |
|---|---|---|---|---|
| OAT[c] | Ornithine--oxo-acid aminotransferase | Orn[c] + aKG[c] <-> Glu[c] + L-Glut-gsAld[c] | Proline metabolism | 2.6.1.13 |
| TSB2[p] | Tryptophan synthase | Indole[p] + Ser[p] -> Trp[p] + H2O[p] | Tryptophan biosynthesis | 4.2.1.20 |
| ADT[p] | arogenate dehydratase | Arogenate[p] + H+[p] -> Phe[p] + H2O[p] + CO2[p] | Phenylalanine biosynthesis | 4.2.1.91 |
| LDH[c] | L-lactate dehydrogenase | NADH[c] + Pyr[c] + H+[c] -> NAD+[c] + Lactate[c] | Fermentation | 1.1.1.27 |
| MMSDH[c] | malonate-semialdehyde dehydrogenase (acetylating) | NADP+[c] + Mal-sAld[c] + CoA[c] -> Ace-CoA[c] + NADPH[c] + CO2[c] | Propanoate Metabolism | 1.2.1.18 |
| IDH[m] | Isocitrate dehydrogenase (NAD+) | Isocit[m] + NAD+[m] -> aKG[m] + CO2[m] + NADH[m] | TCA Cycle | 1.1.1.41 |
| ALDH1[c] | aldehyde dehydrogenase | Acetald[c] + NAD+[c] + H2O[c] -> NADH[c] + Acetate[c] + 2 H+[c] | Fermentation | 1.2.1.3 |
| ALDH1[p] | aldehyde dehydrogenase | Acetald[p] + NAD+[p] + H2O[p] -> NADH[p] + Acetate[p] + 2 H+[c] | Fermentation | 1.2.1.3 |
| SSADH1[m] | Succinate-semialdehyde dehydrogenase | H2O[m] + NAD+[m] + SuccsAld[m] -> NADH[m] + Succ[m] + 2 H+[c] | GABA metabolism | 1.2.1.24 |
| TYRAAT[p] | arogenate dehydrogenase | Arogenate[p] + NADP+[p] -> Tyr[p] + NADPH[p] + CO2[p] | Tyrosine biosynthesis | 1.3.1.78 |
| THRALD[p] | L-threonine aldolase | Thr[p] -> Gly[p] + Acetald[p] | Glycine biosyntheiss | 4.1.2.5 |
| ADH[c] | alcohol dehydrogenase | Acetald[c] + NADH[c] + H+[c] <-> Ethanol[c] + NAD+[c] | Fermentation | 1.1.1.1 |
| FBP1[c] | Fructose-bisphosphatase | Fruct16bP[c] + H2O[c] -> Fruct6P[c] + pi[c] | Glycolysis/Gluconeogensis | 3.1.3.11 |
| FBP1[p] | Fructose-bisphosphatase | Fruct16bP[p] + H2O[p] -> Fruct6P[p] + pi[p] | Glycolysis/Gluconeogensis | 3.1.3.11 |
| SPS[c] | Sucrose-phosphate synthase | UDP-Glc[c] + Fruct6P[c] -> Sucr6P[c] + UDP[c] | Sucrose metabolism | 2.4.1.14 |
| PGI[c] | Glucose-6-phosphate isomerase | b-Glc6P[c] <-> Fruct6P[c] | Sucrose metabolism | 5.3.1.9 |
| PGI[p] | Glucose-6-phosphate isomerase | b-Glc6P[p] <-> Fruct6P[p] | Glycolysis/Gluconeogensis | 5.3.1.9 |
| CIN[c] | Beta-fructofuranosidase | Sucrose[c] + H2O[c] -> Fruct[c] + a-Glc[c] | Sucrose metabolism | 3.2.1.26 |
| SPP[c] | Sucrose-phosphatase | Sucr6P[c] + H2O[c] -> Sucrose[c] + pi[c] | Sucrose metabolism | 3.1.3.24 |
| SUS[c] | Sucrose synthase | UDP-Glc[c] + Fruct[c] <-> Sucrose[c] + UDP[c] | Sucrose metabolism | 2.4.1.13 |
| INO1[c] | myo-inositol-1-phosphate synthase | b-Glc6P[c] -> D-myo3mP[c] | Cell wall metabolism | 5.5.1.4 |

| | | | | |
|---|---|---|---|---|
| SIR[p] | Sulfite reductase (ferredoxin) | sulfite[p] + 3 redferr[p] + 8 H+[p] -> sulfide[p] + 3 oxiferr[p] + 3 H2O[p] | Sulfate assimilation | 1.8.7.1 |
| RCS[p] | Cysteine synthase | O-Ace-L-ser[p] + sulfide[p] -> Cys[p] + Acetate[p] + H+[p] | Cysteine biosynthesis | 2.5.1.47 |
| FTHFL[c] | Formate--tetrahydrofolate ligase | ATP[c] + Formate[c] + THF[c] -> ADP[c] + pi[c] + N10FormTHF[c] | Folates metabolism | 6.3.4.3 |
| FTHFL[m] | Formate--tetrahydrofolate ligase | ATP[m] + Formate[m] + THF[m] -> ADP[m] + pi[m] + N10FormTHF[m] | Folates metabolism | 6.3.4.3 |
| FTHFL[p] | Formate--tetrahydrofolate ligase | ATP[p] + Formate[p] + THF[p] -> ADP[p] + pi[p] + N10FormTHF[p] | Folates metabolism | 6.3.4.3 |
| PURU[c] | Formyltetrahydrofolate deformylase | H2O[c] + N10FormTHF[c] -> THF[c] + Formate[c] + H+[c] | Folates metabolism | 3.5.1.10 |
| PURU[m] | Formyltetrahydrofolate deformylase | H2O[m] + N10FormTHF[m] -> THF[m] + Formate[m] + H+[m] | Folates metabolism | 3.5.1.10 |
| PURU[p] | Formyltetrahydrofolate deformylase | H2O[p] + N10FormTHF[p] -> THF[p] + Formate[p] + H+[p] | Folates metabolism | 3.5.1.10 |
| SHM1[c] | Glycine hydroxymethyltransferase | 510MeteTHF[c] + Gly[c] + H2O[c] <-> Ser[c] + THF[c] | Glycine biosyntheiss | 2.1.2.1 |
| SHM1[m] | Glycine hydroxymethyltransferase | 510MeteTHF[m] + Gly[m] + H2O[m] <-> Ser[m] + THF[m] | Glycine biosynthesis | 2.1.2.1 |
| SHM1[p] | Glycine hydroxymethyltransferase | 510MeteTHF[p] + Gly[p] + H2O[p] <-> Ser[p] + THF[p] | Glycine biosyntheiss | 2.1.2.1 |
| APS[p] | Glucose-1-phosphate adenylyltransferase | a-Glc1P[p] + ATP[p] + H+[p] -> ADP-D-Glu[p] + ppi[p] | Starch metabolism | 2.7.7.27 |
| UMPS[p] | Orotidine-5'-phosphate decarboxylase | O5P[p] + H+[p] -> CO2[p] + UMP[p] | Pyrimidine metabolism | 4.1.1.23 |
| ASA[p] | Anthranilate synthase | Chorismate[p] + Gln[p] -> Glu[p] + Anth[p] + Pyr[p] + H+[p] | Tryptophan biosynthesis | 4.1.3.27 |
| TD[p] | Threonine dehydratase | Thr[p] -> 2oxoBut[p] + ammonia[p] + H+[p] | Isoleucine biosynthesis | 4.3.1.19 |
| TPI[c] | Triosephosphate isomerase | G3P[c] <-> DHAP[c] | Glycolysis/Gluconeogensis | 5.3.1.1 |
| TPI[p] | Triosephosphate isomerase | G3P[p] <-> DHAP[p] | Glycolysis/Gluconeogensis | 5.3.1.1 |
| PRS[p] | ribose-phosphate diphosphokinase | ATP[p] + R5P[p] -> PRpi[p] + AMP[p] + H+[p] | Purine metabolism | 2.7.6.1 |

| | | | | |
|---|---|---|---|---|
| RPI[c] | Ribose 5-phosphate epimerase | R5P[c] <-> Ru5P[c] | Pentose Phosphate Pathway | 5.3.1.6 |
| RPI[p] | Ribose 5-phosphate epimerase | R5P[p] <-> Ru5P[p] | Pentose Phosphate Pathway | 5.3.1.6 |
| GAPN[c] | Glyceraldehyde-3-phosphate dehydrogenase (NADP+) | G3P[c] + NADP+[c] + H2O[c] -> 3PG[c] + NADPH[c] + 2 H+[c] | Glycolysis/Gluconeogensis | 1.2.1.9 |
| GAPDH[c] | Glyceraldehyde 3-phosphate dehydrogenase (phosphorylating) | G3P[c] + pi[c] + NAD+[c] <-> 1,3-DPGA[c] + NADH[c] + H+[c] | Glycolysis/Gluconeogensis | 1.2.1.12 |
| GAPDH[p] | Glyceraldehyde 3-phosphate dehydrogenase (phosphorylating) | G3P[p] + pi[p] + NAD+[p] <-> 1,3-DPGA[p] + NADH[p] + H+[p] | Glycolysis/Gluconeogensis | 1.2.1.12 |
| ALD[c] | Fructose-bisphosphate aldolase | Fruct16bP[c] <-> DHAP[c] + G3P[c] | Glycolysis/Gluconeogensis | 4.1.2.13 |
| ALD[p] | Fructose-bisphosphate aldolase | Fruct16bP[p] <-> DHAP[p] + G3P[p] | Glycolysis/Gluconeogensis | 4.1.2.13 |
| ATPPRT[p] | ATP phosphoribosyltransferase | ATP[p] + PRpi[p] -> Pr-ATP[p] + ppi[p] | Histidine biosynthesis | 2.4.2.17 |
| AMPRT[p] | Anthranilate phosphoribosyltransferase | Anth[p] + PRpi[p] -> N5PAnth[p] + ppi[p] | Tryptophan biosynthesis | 2.4.2.18 |
| FUM[m] | Fumarate hydratase | Malate[m] <-> Fumr[m] + H2O[m] | TCA Cycle | 4.2.1.2 |
| ADNSL[p] | Adenylosuccinate lyase | Ade-Succ[p] -> Fumr[p] + AMP[p] | Purine metabolism | 4.3.2.2 |
| ARGSL[c] | Argininosuccinate lyase | ArgSucc[c] -> Arg[c] + Fumr[c] | Arginine biosynthesis | 4.3.2.1 |
| BCAT1[p] | Branched-chain amino acid aminotransferase | Leu[p] + aKG[p] <-> Glu[p] + 2kiCapr[p] | Leucine biosynthesis | 2.6.1.42 |
| IMPC[p] | IMP cyclohydrolase | Pr-FormCarb[p] -> IMP[p] + H2O[p] | Purine metabolism | 3.5.4.10 |
| PURA1[p] | adenylosuccinate synthetase | Asp[p] + IMP[p] + GTP[p] -> Ade-Succ[p] + pi[p] + GDP[p] + 2 H+[p] | Purine metabolism | 6.3.4.4 |
| AMETHM[c] | S-adenosyl-L-methionine:L-histidine N-methyltransferase | S-Ade-L-meth[c] <-> S-Ade-L-H[c] | Methionine biosynthesis | 2.1.1.- |
| HDH1[p] | Histidinol dehydrogenase | His-al[p] + NAD+[p] + H2O[p] -> His[p] + NADH[p] + 2 H+[p] | Histidine biosynthesis | 1.1.1.23 |

| | | | | |
|---|---|---|---|---|
| MIOX[c] | Myo-inositol oxygenase | O2[c] + m-Ino[c] -> Glucuronate[c] + H2O[c] + H+[c] | Cell wall metabolism | 1.13.99.1 |
| MIOMP[c] | Myo-inositol-1(or 4)-monophosphatase | D-myo3mP[c] + H2O[c] -> m-Ino[c] + pi[c] | Cell wall metabolism | 3.1.3.25 |
| FNR[p] | Ferredoxin--NADP(+) reductase | 2 redferr[p] + NADP+[p] + H+[p] <-> 2 oxiferr[p] + NADPH[p] | GS-GOGAT Cycle | 1.18.1.2 |
| AKGDH[m] | 2-oxoglutarate synthase | aKG[m] + CoA[m] + NAD+[m] -> Suc-CoA[m] + CO2[m] + NADH[m] | TCA Cycle | 1.2.7.3 |
| DADH[p] | Dihydroxy-acid dehydratase | 23dhiVal[p] -> 2kiVal[p] + H2O[p] | Valine biosynthesis | 4.2.1.9 |
| IPMS[p] | 2-isopropylMalate synthase | 2kiVal[p] + Ace-CoA[p] + H2O[p] -> 2iPropMal[p] + CoA[p] + H+[p] | Leucine biosynthesis | 2.3.3.13 |
| BCAT2[p] | Branched-chain amino acid aminotransferase | Val[p] + aKG[p] <-> Glu[p] + 2kiVal[p] | Valine biosynthesis | 2.6.1.42 |
| MTHFD1[c] | Methylenetetrahydrofolate dehydrogenase (NAD+) | 510MeteTHF[c] + NAD+[c] -> 510MetTHF[c] + NADH[c] | Folates metabolism | 1.5.1.15 |
| MTHFD1[m] | Methylenetetrahydrofolate dehydrogenase (NAD+) | 510MeteTHF[m] + NAD+[m] -> 510MetTHF[m] + NADH[m] | Folates metabolism | 1.5.1.15 |
| MTHFD1[p] | Methylenetetrahydrofolate dehydrogenase (NAD+) | 510MeteTHF[p] + NAD+[p] -> 510MetTHF[p] + NADH[p] | Folates metabolism | 1.5.1.15 |
| MTHFD2[c] | Methylenetetrahydrofolate dehydrogenase (NADP+) | 510MeteTHF[c] + NADP+[c] -> NADPH[c] + 510MetTHF[c] | Folates metabolism | 1.5.1.5 |
| MTHFD2[m] | Methylenetetrahydrofolate dehydrogenase (NADP+) | 510MeteTHF[m] + NADP+[m] -> NADPH[m] + 510MetTHF[m] | Folates metabolism | 1.5.1.5 |
| MTHFD2[p] | Methylenetetrahydrofolate dehydrogenase (NADP+) | 510MeteTHF[p] + NADP+[p] -> NADPH[p] + 510MetTHF[p] | Folates metabolism | 1.5.1.5 |
| GDC[m] | Ser hydroxymethyl transferase | Gly[m] + THF[m] + NAD+[m] -> ammonia[m] + 510MeteTHF[m] + CO2[m] + NADH[m] + H+[m] | Photorespiration | 1.4.4.2, 1.8.1.4, 2.1.2.10 |
| PROC1[c] | Pyrroline-5-carboxylate reductase | Pyrr5Carb[c] + NADH[c] + 2 H+[c] -> Pro[c] + NAD+[c] | Proline metabolism | 1.5.1.2 |
| PROC1[p] | Pyrroline-5-carboxylate reductase | Pyrr5Carb[p] + NADH[p] + 2 H+[p] -> Pro[p] + NAD+[p] | Proline metabolism | 1.5.1.2 |

| | | | | |
|---|---|---|---|---|
| PROC2[c] | Pyrroline-5-carboxylate reductase | Pyrr5Carb[c] + NADPH[c] + 2 H+[c] -> Pro[c] + NADP+[c] | Proline metabolism | 1.5.1.2 |
| PROC2[p] | Pyrroline-5-carboxylate reductase | Pyrr5Carb[p] + NADPH[p] + 2 H+[p] -> Pro[p] + NADP+[p] | Proline metabolism | 1.5.1.2 |
| CBL[p] | Cystathionine beta-lyase | cysttn[p] + H2O[p] -> ammonia[p] + Pyr[p] + HomoCys[p] + H+[p] | Methionine biosynthesis | 4.4.1.8 |
| ACO2[c] | citrate hydrolyase | c-Aco[c] + H2O[c] <-> Isocit[c] | Glyoxylate Cycle | 4.2.1.3 |
| ACO2[m] | citrate hydrolyase | c-Aco[m] + H2O[m] <-> Isocit[m] | TCA Cycle | 4.2.1.3 |
| GADH[c] | Glycolaldehyde dehydrogenase | H2O[c] + NAD+[c] + Glycoald[c] -> NADH[c] + Glycolate[c] + 2 H+[c] | Folates metabolism | 1.2.1.21 |
| PGP[p] | Phosphoglycolate phosphatase | H2O[p] + 2PhosGlyco[p] -> Glycolate[p] + pi[p] | Photorespiration | 3.1.3.18 |
| GPUT[c] | Glucuronate-1-phosphate uridylyltransferase | a-D-Glucur-1-P[c] + UTP[c] + H+[c] -> UDP-Glucur[c] + ppi[c] | Cell wall metabolism | 2.7.7.44 |
| UDPGDC[c] | UDP-glucuronate decarboxylase | UDP-Glucur[c] + H+[c] -> UDP-Xyl[c] + CO2[c] | Cell wall metabolism | 4.1.1.35 |
| HPR[c] | Hydroxypyruvate reductase | Hydpyr[c] + NADH[c] + H+[c] -> Glycerate[c] + NAD+[c] | Photorespiration | 1.1.1.29 |
| ATC[p] | Aspartate carbamoyltransferase | Asp[p] + CarmteP[p] -> N-CarbL-Asp[p] + pi[p] + H+[p] | Pyrimidine metabolism | 2.1.3.2 |
| OTC[c] | Ornithine carbamoyltransferase | Orn[c] + CarmteP[c] <-> citrulline[c] + pi[c] + H+[c] | Proline metabolism | 2.1.3.3 |
| TS[p] | Threonine synthase | O-P-L-hmser[p] + H2O[p] -> pi[p] + Thr[p] | Threonine biosynthesis | 4.2.3.1 |
| UEL[c] | UDP-arabinose 4-epimerase | UDP-Xyl[c] -> UDP-L-arab[c] | Cell wall metabolism | 5.1.3.5 |
| GLCAK[c] | Glucuronokinase | Glucuronate[c] + ATP[c] -> a-D-Glucur-1-P[c] + ADP[c] + H+[c] | Cell wall metabolism | 2.7.1.43 |
| PGK[c] | Phosphoglycerate kinase | 3PG[c] + ATP[c] <-> 1,3-DPGA[c] + ADP[c] | Glycolysis/Gluconeogensis | 2.7.2.3 |
| PGK[p] | Phosphoglycerate kinase | 3PG[p] + ATP[p] <-> 1,3-DPGA[p] + ADP[p] | Glycolysis/Gluconeogensis | 2.7.2.3 |
| PGDH[p] | Phosphoglycerate dehydrogenase | 3PG[p] + NAD+[p] -> 3Phoshpyr[p] + NADH[p] + H+[p] | Serine biosynthesis | 1.1.1.95 |
| GLYK[p] | Glycerate kinase | Glycerate[p] + ATP[p] -> 3PG[p] + ADP[p] + H+[p] | Photorespiration | 2.7.1.31 |
| PGLYCM[c] | Phosphoglycerate mutase | 3PG[c] <-> 2PG[c] | Glycolysis/Gluconeogensis | 5.4.2.1 |

| | | | | |
|---|---|---|---|---|
| PGLYCM[p] | Phosphoglycerate mutase | 3PG[p] <-> 2PG[p] | Glycolysis/Gluconeogensis | 5.4.2.1 |
| PRK[p] | Phosphoribulokinase | Ru5P[p] + ATP[p] -> RuBP[p] + ADP[p] + H+[p] | Calvin cycle | 2.7.1.19 |
| G6PGH1[c] | Phosphogluconate dehydrogenase (decarboxylating) | 6-P-gluco[c] + NADP+[c] -> Ru5P[c] + CO2[c] + NADPH[c] | Pentose Phosphate Pathway | 1.1.1.44 |
| G6PGH2[p] | Phosphogluconate dehydrogenase (decarboxylating) | 6-P-gluco[p] + NADP+[p] -> Ru5P[p] + CO2[p] + NADPH[p] | Pentose Phosphate Pathway | 1.1.1.44 |
| RPE[c] | ribulose phosphate 3-epimerase | Ru5P[c] <-> Xu5P[c] | Pentose Phosphate Pathway | 5.1.3.1 |
| RPE[p] | ribulose phosphate 3-epimerase | Ru5P[p] <-> Xu5P[p] | Pentose Phosphate Pathway | 5.1.3.1 |
| HPDH[c] | 3-hydroxypropionate dehydrogenase | NAD+[c] + 3hprpnte[c] -> NADH[c] + Mal-sAld[c] + H+[c] | Propanoate Metabolism | 1.1.1.59 |
| TKT1[c] | Transketolase | R5P[c] + Xu5P[c] <-> D-Sed-7P[c] + G3P[c] | Pentose Phosphate Pathway | 2.2.1.1 |
| TKT1[p] | Transketolase | R5P[p] + Xu5P[p] <-> D-Sed-7P[p] + G3P[p] | Pentose Phosphate Pathway | 2.2.1.1 |
| GABA-TK[m] | 4-aminobutyrate aminotransferase (GABA-TK) | GABA[m] + aKG[m] -> Glu[m] + SuccsAld[m] | GABA metabolism | 2.6.1.19 |
| GABA-TP[m] | 4-aminobutyrate aminotransferase (GABA-TP) | GABA[m] + Pyr[m] -> Ala[m] + SuccsAld[m] | GABA metabolism | 2.6.1.19 |
| LB1[p] | leucine biosynthesis | 2iProp3Succ[p] -> 2kiCapr[p] + CO2[p] | Leucine biosynthesis | |
| MTHFC[m] | Methenyltetrahydrofolate cyclohydrolase | H2O[m] + 510MetTHF[m] <-> N10FormTHF[m] + H+[m] | Folates metabolism | 3.5.4.9 |
| PDHE1[p] | Pyruvate dehydrogenase (lipoamide) | Pyr[p] + pyrdeh1[p] + H+[p] <-> pyrdeh2[p] + CO2[p] | Pyruvate dehydrogenase complex | 1.2.4.1 |
| EMB[p] | Chorismate synthase | 5EnoShiki3P[p] -> pi[p] + Chorismate[p] | Tryptophan biosynthesis | 4.2.3.5 |
| CM[p] | Chorismate mutase | Chorismate[p] -> Prep[p] | Phenylalanine biosynthesis | 5.4.99.5 |
| HSK[p] | Homoserine kinase | HomoSer[p] + ATP[p] -> O-P-L-hmser[p] + ADP[p] + H+[p] | Threonine biosynthesis | 2.7.1.39 |

| | | | | |
|---|---|---|---|---|
| HSDH1[p] | Homoserine dehydrogenase | L-Asp-sAld[p] + NADH[p] + H+[p] -> HomoSer[p] + NAD+[p] | Threonine biosynthesis | 1.1.1.3 |
| HSDH2[p] | Homoserine dehydrogenase | L-Asp-sAld[p] + NADPH[p] + H+[p] -> HomoSer[p] + NADP+[p] | Threonine biosynthesis | 1.1.1.3 |
| HXK[c] | hexokinase | a-Glc[c] + ATP[c] -> a-Glc6P[c] + ADP[c] + H+[c] | Sucrose metabolism | 2.7.1.1 |
| HXK[p] | hexokinase | a-Glc[p] + ATP[p] -> a-Glc6P[p] + ADP[p] + H+[p] | Starch metabolism | 2.7.1.1 |
| PHS[p] | starch phosphorylase | glucan[p] + pi[p] -> a-Glc1P[p] | Starch metabolism | 2.4.1.1 |
| DAHPS[p] | 2-dehydro-3-deoxyphosphoheptonate aldolase | PEP[p] + D-ery4P[p] + H2O[p] -> 3DODara7P[p] + pi[p] | Tryptophan biosynthesis | 2.5.1.54 |
| TALA[c] | Transaldolase | G3P[c] + D-Sed-7P[c] -> Fruct6P[c] + D-ery4P[c] | Pentose Phosphate Pathway | 2.2.1.2 |
| TALA[p] | Transaldolase | G3P[p] + D-Sed-7P[p] -> Fruct6P[p] + D-ery4P[p] | Pentose Phosphate Pathway | 2.2.1.2 |
| SBPGL[p] | Sedoheptulose 1,7-bisphosphate D-glyceraldehyde-3-phosphate-lyase | DHAP[p] + D-ery4P[p] -> D-Sed-17bP[p] | Calvin cycle | 4.1.2.- |
| TKT2[c] | Transketolase | D-ery4P[c] + Xu5P[c] <-> Fruct6P[c] + G3P[c] | Pentose Phosphate Pathway | 2.2.1.1 |
| TKT2[p] | Transketolase | D-ery4P[p] + Xu5P[p] <-> Fruct6P[p] + G3P[p] | Pentose Phosphate Pathway | 2.2.1.1 |
| SBP[p] | Sedoheptulose-bisphosphatase | H2O[p] + D-Sed-17bP[p] -> pi[p] + D-Sed-7P[p] | Calvin cycle | 3.1.3.37 |
| OMPD[p] | Orotate phosphoribosyltransferase | PRpi[p] + Orot[p] -> O5P[p] + ppi[p] | Pyrimidine metabolism | 2.4.2.10 |
| ACO1[c] | Aconitate hydratase | Citrate[c] <-> c-Aco[c] + H2O[c] | Glyoxylate Cycle | 4.2.1.3 |
| ACO1[m] | isocitrate hydrolyase | Citrate[m] <-> c-Aco[m] + H2O[m] | TCA Cycle | 4.2.1.3 |
| ASS[c] | argininosuccinate synthetase | Asp[c] + citrulline[c] + ATP[c] -> ArgSucc[c] + ppi[c] + AMP[c] + H+[c] | Arginine biosynthesis | 6.3.4.5 |
| DHO[p] | Dihydroorotase | N-CarbL-Asp[p] + H+[p] -> DHooro[p] + H2O[p] | Pyrimidine metabolism | 3.5.2.3 |
| 6PGL[c] | 6-phosphogluconolactonase | D-Glu-d-Lac6P[c] + H2O[c] -> 6-P-gluco[c] + H+[c] | Pentose Phosphate Pathway | 3.1.1.31 |

| | | | | |
|---|---|---|---|---|
| 6PGL[p] | 6-phosphogluconolactonase | D-Glu-d-Lac6P[p] + H2O[p] -> 6-P-gluco[p] + H+[p] | Pentose Phosphate Pathway | 3.1.1.31 |
| SBE[p] | 1,4-alpha-glucan branching enzyme | glucan[p] -> Starch[p] + H2O[p] | Starch metabolism | 2.4.1.18 |
| CCOR[m] | Q-cytochrome c oxidoreductase (complex III) | 2 Cyto-Oxi[m] + QH2[m] + 1.5 H+[m] -> 2 Cyto-Red[m] + Q[m] + 1.5 H+[c] | Oxidative phosphorylation | 1.10.2.2 |
| NAD9[m] | NADH-coenzyme Q oxidoreductase (complex I) | Q[m] + NADH[m] + H+[m] -> QH2[m] + NAD+[m] | Oxidative phosphorylation | 1.6.5.3 |
| SDH[m] | Succinate-Q oxidoreductase (complex II) | Q[m] + Succ[m] -> QH2[m] + Fumr[m] | TCA Cycle | 1.3.5.1 |
| BCAT3[p] | Branched-chain amino acid aminotransferase | Ile[p] + aKG[p] <-> Glu[p] + 2k3MetVal[p] | Isoleucine biosynthesis | 2.6.1.42 |
| AGS[c] | Glutamate N-acetyltransferase | Glu[c] + NAce-L-Orn[c] -> N-Ace-L-glutt[c] + Orn[c] | Arginine biosynthesis | 2.3.1.35 |
| AOAT[c] | Acetylornithine aminotransferase | Glu[c] + N-A-L-gluttsemald[c] -> NAce-L-Orn[c] + aKG[c] | Arginine biosynthesis | 2.6.1.11 |
| ASAD[p] | Aspartate-semialdehyde dehydrogenase | NADPH[p] + Asp4P[p] + H+[p] -> NADP+[p] + pi[p] + L-Asp-sAld[p] | Threonine biosynthesis | 1.2.1.11 |
| DHDPS[p] | dihydrodipicolinate synthase | Pyr[p] + L-Asp-sAld[p] -> 2 H2O[p] + L-23-DHDC[p] + H+[p] | Lysine biosynthesis | 4.3.3.7 |
| TSA[p] | Indole-3-glycerol-phosphate lyase | ind3Ace-GP[p] -> Indole[p] + G3P[p] | Tryptophan biosynthesis | 4.1.2.8 |
| SK[p] | shikimate-kinase | Shikimate[p] + ATP[p] -> Shikm3P[p] + ADP[p] + H+[p] | Tryptophan biosynthesis | 2.7.1.71 |
| SKDH[p] | Shikimate 5-dehydrogenase | NADPH[p] + 3DHShiki[p] + H+[p] -> NADP+[p] + Shikimate[p] | Tryptophan biosynthesis | 1.1.1.25 |
| SS[p] | Starch synthase | ADP-D-Glu[p] -> ADP[p] + glucan[p] + H+[p] | Starch metabolism | 2.4.1.21 |
| PDHE2[p] | Dihydrolipoamide S-acetyltransferase | CoA[p] + pyrdeh2[p] <-> Ace-CoA[p] + pyrdeh3[p] | Pyruvate dehydrogenase complex | 2.3.1.12 |
| NAGK[c] | Acetylglutamate kinase | N-Ace-L-glutt[c] + ATP[c] -> NAceGlutP[c] + ADP[c] | Arginine biosynthesis | 2.7.2.8 |
| MFP[c] | 3-hydroxyacyl-CoA dehydrogenase | AcrCoA[c] + H2O[c] -> 3HOPropCoA[c] | Propanoate Metabolism | 4.2.1.17 |

| | | | | |
|---|---|---|---|---|
| DAPF[p] | Diaminopimelate epimerase | LLdapime[p] -> mPime[p] | Lysine biosynthesis | 5.1.1.7 |
| EPI[c] | Glucose-6 phosphate 1-epimerase | a-Glc6P[c] <-> b-Glc6P[c] | Sucrose metabolism | 5.1.3.15 |
| EPI[p] | Glucose-6 phosphate 1-epimerase | a-Glc6P[p] <-> b-Glc6P[p] | Starch metabolism | 5.1.3.15 |
| CESA1[c] | Cellulose synthase (UDP-forming) | UDP-Glc[c] -> cellulose[c] + UDP[c] + H+[c] | Cell wall metabolism | 2.4.1.12 |
| HDH2[p] | Histidinol dehydrogenase | His-ol[p] + NAD+[p] -> His-al[p] + NADH[p] + H+[p] | Histidine biosynthesis | 1.1.1.23 |
| IMPL2[p] | Histidinol-phosphatase | L-HisP[p] + H2O[p] -> His-ol[p] + pi[p] | Histidine biosynthesis | 3.1.3.15 |
| KARI[p] | Ketol-acid reductoisomerase | 2Acelac[p] + NADPH[p] + H+[p] -> 23dhiVal[p] + NADP+[p] | Valine biosynthesis | 1.1.1.86 |
| DHPS[m] | Dihydropteroate synthase | p-Abenz[m] + 2a4h6hm78dP[m] -> 78dhPte[m] + ppi[m] | Folates metabolism | 2.5.1.15 |
| DHQS[p] | 3-dehydroquinate synthase | 3DODara7P[p] -> 3DHQuin[p] + pi[p] | Tryptophan biosynthesis | 4.2.3.4 |
| DHQDH[p] | 3-dehydroquinate dehydratase | 3DHQuin[p] -> H2O[p] + 3DHShiki[p] | Tryptophan biosynthesis | 4.2.1.10 |
| RBCS-O[p] | Ribulose-bisphosphate oxygenase | RuBP[p] + O2[p] -> 2PhosGlyco[p] + 3PG[p] + 2 H+[p] | photorespiration | 4.1.1.39 |
| CHY[c] | 3-hydroxyisobutyryl-CoA hydrolase | 3HOPropCoA[c] + pi[c] + ADP[c] -> 3hprpnte[c] + CoA[c] + ATP[c] | Propanoate Metabolism | 3.1.2.4 |
| HPA[p] | Histidinol-phosphate aminotransferase | iaP[p] + Glu[p] -> L-HisP[p] + aKG[p] | Histidine biosynthesis | 2.6.1.9 |
| P5CS2[c] | Glutamate-5-semialdehyde dehydrogenase | L-Glut-5P[c] + NADPH[c] + H+[c] -> L-Glut-gsAld[c] + pi[c] + NADP+[c] | Proline metabolism | 1.2.1.41 |
| P5CS2[p] | Glutamate-5-semialdehyde dehydrogenase | L-Glut-5P[p] + NADPH[p] + H+[p] -> L-Glut-gsAld[p] + pi[p] + NADP+[p] | Proline metabolism | 1.2.1.41 |
| PR2[c] | Proline biosynthesis | L-Glut-gsAld[c] <-> H2O[c] + Pyrr5Carb[c] | Proline metabolism | |
| PR1[p] | Proline biosynthesis | L-Glut-gsAld[p] <-> H2O[p] + Pyrr5Carb[p] | Proline metabolism | |
| AGPR[c] | N-acetyl-gamma-glutamyl-phosphate reductase | NAceGlutP[c] + NADPH[c] + H+[c] -> N-A-L-gluttsemald[c] + NADP+[c] + pi[c] | Arginine biosynthesis | 1.2.1.38 |
| IGPD[p] | Imidazoleglycerol-phosphate dehydratase | D-eryigP[p] -> iaP[p] + H2O[p] | Histidine biosynthesis | 4.2.1.19 |

| | | | | |
|---|---|---|---|---|
| EPSPS[p] | 3-phosphoshikimate 1-carboxyvinyltransferase | Shikm3P[p] + PEP[p] -> 5EnoShiki3P[p] + pi[p] | Tryptophan biosynthesis | 2.5.1.19 |
| IGPS[p] | indole-3-glycerol-phosphate synthase | 1-ocdoP[p] + H+[p] -> ind3Ace-GP[p] + CO2[p] + H2O[p] | Tryptophan biosynthesis | 4.1.1.48 |
| PAI[p] | Phosphoribosylanthranilate isomerase | N5PAnth[p] -> 1-ocdoP[p] | Tryptophan biosynthesis | 5.3.1.24 |
| IIL[p] | 3-isopropylMalate dehydratase | 2iPropMal[p] + H2O[p] <-> 3iPropMal[p] | Leucine biosynthesis | 4.2.1.33 |
| PRAPH[p] | Phosphoribosyl-ATP pyrophosphatase | Pr-ATP[p] + H2O[p] -> Pr-AMP[p] + ppi[p] + H+[p] | Histidine biosynthesis | 3.6.1.31 |
| PRACH[p] | Phosphoribosyl-AMP cyclohydrolase | Pr-AMP[p] + H2O[p] -> Pr-FormCarbP[p] | Histidine biosynthesis | 3.5.4.19 |
| PSAT[p] | Phosphoserine aminotransferase | Glu[p] + 3Phoshpyr[p] -> 3PhosSer[p] + aKG[p] | Serine biosynthesis | 2.6.1.52 |
| DHPR1[p] | Dihydrodipicolinate reductase | L-23-DHDC[p] + NADH[p] + H+[p] -> Thdpico[p] + NAD+[p] | Lysine biosynthesis | 1.3.1.26 |
| DHPR2[p] | Dihydrodipicolinate reductase | L-23-DHDC[p] + NADPH[p] + H+[p] -> Thdpico[p] + NADP+[p] | Lysine biosynthesis | 1.3.1.26 |
| IMDH[p] | 3-isopropylMalate dehydrogenase | 3iPropMal[p] + NAD+[p] -> 2iProp3Succ[p] + NADH[p] + H+[p] | Leucine biosynthesis | 1.1.1.85 |
| PCOR[c] | Propanoyl-CoA: 2,3-oxidoreductase | PropCoA[c] + NAD+[c] -> AcrCoA[c] + NADH[c] | Propanoate Metabolism | 1.3.99.3 |
| IGPS2[p] | Imidazole glycerol phosphate synthase | Pr-BulSylFormP[p] + Gln[p] -> Glu[p] + D-eryigP[p] + AICAR[p] + H+[p] | Histidine biosynthesis | 2.4.2.- |
| PRACFT[p] | Phosphoribosylaminoimidazolecarboxamide formyltransferase | N10FormTHF[p] + AICAR[p] -> THF[p] + Pr-FormCarb[p] | Purine metabolism | 2.1.2.3 |
| HIS1[p] | N-(5'-phospho-D-ribosylformimino)-5-amino-1-(5''-phosphoribosyl)-4- imidazole carboxamide isomerase | Pr-FormCarbP[p] -> Pr-BulSylFormP[p] | Histidine biosynthesis | 5.3.1.16 |
| KARI2[p] | Ketol-acid reductoisomerase | 2a2hButyr[p] + NADPH[p] + H+[p] -> 23dh3MetVal[p] + NADP+[p] | Isoleucine biosynthesis | 1.1.1.86 |
| ILVD[p] | Dihydroxy-acid dehydratase | 23dh3MetVal[p] -> 2k3MetVal[p] + H2O[p] | Isoleucine biosynthesis | 4.2.1.9 |
| DPE[p] | disproportionating enzyme (D-enzyme) | glucan[p] + H2O[p] -> a-Glc[p] | Starch metabolism | 2.4.1.25 |

| | | | | |
|---|---|---|---|---|
| APR1[p] | Adenylyl-sulfate reductase (glutathione) | Ade-5P[p] + 2 Gluta[p] -> sulfite[p] + AMP[p] + GludiS[p] + 2 H+[p] | Sulfate assimilation | 1.8.4.9 |
| MTHFR2[c] | Methylenetetrahydrofolate reductase (NADPH) | 510MeteTHF[c] + NADH[c] + H+[c] <-> 5MetTHF[c] + NAD+[c] | Folates metabolism | 1.5.1.20 |
| MTHFR2[p] | Methylenetetrahydrofolate reductase (NADPH) | 510MeteTHF[p] + NADPH[p] + H+[p] <-> 5MetTHF[p] + NADP+[p] | Folates metabolism | 1.5.1.20 |
| FRDR[p] | Ferredoxin--NADP(+) reductase | 2 redferr[p] + NAD+[p] + H+[p] <-> 2 oxiferr[p] + NADH[p] | GS-GOGAT Cycle | 1.18.1.3 |
| PAT[p] | Prephenate aminotransferase | Prep[p] + Glu[p] -> Arogenate[p] + aKG[p] | Phenylalanine biosynthesis | 2.6.1.79 |
| AGD2[p] | LL-2,6-diaminoheptanedioate aminotransferase | Thdpico[p] + Glu[p] + H2O[p] + H+[p] -> LLdapime[p] + aKG[p] | Lysine biosynthesis | 2.6.1.83 |
| CPS[p] | carbamoyl-phosphate synthetase (glutamine-hydrolysing) | 2 ATP[p] + Gln[p] + CO2[p] + 2 H2O[p] -> Glu[p] + 2 ADP[p] + pi[p] + CarmteP[p] + 2 H+[p] | Pyrimidine metabolism | 6.3.5.5 |
| PDHE3[p] | dihydrolipoyl dehydrogenase | pyrdeh3[p] + NAD+[p] <-> pyrdeh1[p] + NADH[p] + H+[p] | Pyruvate dehydrogenase complex | 1.8.1.4 |
| PGM[c] | Phosphoglucomutase | a-Glc1P[c] <-> a-Glc6P[c] | Sucrose metabolism | 5.4.2.2 |
| PGM[p] | Phosphoglucomutase | a-Glc1P[p] <-> a-Glc6P[p] | Starch metabolism | 5.4.2.2 |
| ALS2[p] | Acetolactate synthase | Pyr[p] + 2oxoBut[p] + H+[p] -> 2a2hButyr[p] + CO2[p] | Isoleucine biosynthesis | 2.2.1.6 |
| ILEDG[c] | isoleuciene degradation, lumped | CoA[c] + aKG[c] + Ile[c] -> PropCoA[c] + Ace-CoA[c] + Glu[c] | isoleuciene degradation | |
| PSLR[p] | Photosynthesis light reaction | 8 photon[p] + 2 H2O[p] + 3 ADP[p] + 3 pi[p] + 2 NADP+[p] -> O2[p] + 3 ATP[p] + 2 H+[p] + 2 NADPH[p] | Photosynthesis, light reaction | |
| CS[p] | Cystathionine gamma-synthase | Cys[p] + O-P-L-hmser[p] -> cysttn[p] + pi[p] + H+[p] | Methionine biosynthesis | 2.5.1.- |
| DHOX[m] | Dihydroorotate oxidase | O2[m] + DHooro[m] -> H2O2[m] + Orot[m] | Pyrimidine metabolism | 1.3.3.1 |
| DHOD[c] | Dihydroorotate dehydrogenase | NAD+[c] + DHooro[c] -> NADH[c] + Orot[c] + H+[c] | Pyrimidine metabolism | 1.3.5.2 |
| CA[p] | carbonic anhydrase | CO2[p] + H2O[p] -> HCO3[p] | Lipid Biosynthesis | 4.2.1.1 |
| CA[c] | carbonic anhydrase | CO2[c] + H2O[c] -> HCO3[c] | Lipid Biosynthesis | 4.2.1.1 |
| ACC[p] | Acetyl-CoA carboxylase | ATP[p] + Ace-CoA[p] + HCO3[p] -> ADP[p] + pi[p] + Mal-CoA[p] + H+[p] | Lipid Biosynthesis | 6.4.1.2 |

| | | | | |
|---|---|---|---|---|
| GPD1[c] | Glycerol-3-phosphate dehydrogenase (NAD(P)+) | DHAP[c] + NADH[c] + H+[c] <-> SGly3P[c] + NAD+[c] | Lipid Biosynthesis | 1.1.1.8/1.1.1.94 |
| GPD2[c] | Glycerol-3-phosphate dehydrogenase (NAD(P)+) | DHAP[c] + NADPH[c] + H+[c] <-> SGly3P[c] + NADP+[c] | Lipid Biosynthesis | 1.1.1.8/1.1.1.94 |
| FAS160[p] | Fatty acid synthase (n-C16:0), lumped | 21 H+[p] + 7 Mal-CoA[p] + 14 NADPH[p] + Ace-CoA[p] -> 7 CO2[p] + 7 CoA[p] + 7 H2O[p] + 14 NADP+[p] + Palmt[p] | Lipid Biosynthesis | |
| FAS180[p] | Fatty acid synthase (n-C18:0), lumped | 24 H+[p] + 8 Mal-CoA[p] + 16 NADPH[p] + Ace-CoA[p] -> 8 CO2[p] + 8 CoA[p] + 8 H2O[p] + 16 NADP+[p] + Steat[p] | Lipid Biosynthesis | |
| FASL181[p] | Fatty acid synthase (n-C18:1), lumped | 25 H+[p] + 8 Mal-CoA[p] + 17 NADPH[p] + Ace-CoA[p] -> 8 CO2[p] + 8 CoA[p] + 8 H2O[p] + 17 NADP+[p] + Olet[p] | Lipid Biosynthesis | |
| FASL182[p] | Fatty acid synthase (n-C18:2), lumped | 26 H+[p] + 8 Mal-CoA[p] + 18 NADPH[p] + Ace-CoA[p] -> 8 CO2[p] + 8 CoA[p] + 8 H2O[p] + 18 NADP+[p] + Lolet[p] | Lipid Biosynthesis | |
| FAS183[p] | Fatty acid synthase (n-C18:3), lumped | 27 H+[p] + 8 Mal-CoA[p] + 19 NADPH[p] + Ace-CoA[p] -> 8 CO2[p] + 8 CoA[p] + 8 H2O[p] + 19 NADP+[p] + Lolnt[p] | Lipid Biosynthesis | |
| FACOAL160[c] | fatty-acid--CoA ligase (n-C16:0) | Palmt[c] + ATP[c] + CoA[c] <-> Palm-CoA[c] + AMP[c] + ppi[c] | Lipid Biosynthesis | |
| FACOAL180[c] | fatty-acid--CoA ligase (n-C18:0) | Steat[c] + ATP[c] + CoA[c] <-> Stea-CoA[c] + AMP[c] + ppi[c] | Lipid Biosynthesis | |
| FACOAL181[c] | fatty-acid--CoA ligase (n-C18:1) | Olet[c] + ATP[c] + CoA[c] <-> Ole-CoA[c] + AMP[c] + ppi[c] | Lipid Biosynthesis | |
| FACOAL182[c] | fatty-acid--CoA ligase (n-C18:2) | Lolet[c] + ATP[c] + CoA[c] <-> Lole-CoA[c] + AMP[c] + ppi[c] | Lipid Biosynthesis | |
| FACOAL183[c] | fatty-acid--CoA ligase (n-C18:3) | Lolnt[c] + ATP[c] + CoA[c] <-> Loln-CoA[c] + AMP[c] + ppi[c] | Lipid Biosynthesis | |
| TAGS[c] | Triglyceride synthesis | SGly3P[c] + 0.668 Palm-CoA[c] + 0.045 Stea-CoA[c] + 0.976 Ole-CoA[c] + 1.261 Lole-CoA[c] + 0.048 Loln-CoA[c] + H2O[c] -> TAG[c] + 3 CoA[c] + pi[c] | Lipid Biosynthesis | |
| PAL[c] | Phenylalanine ammonia-lyase | Phe[c] -> ammonia[c] + t-Cinn[c] + H+[c] | Lignin biosynthesis | 4.3.1.25 |
| TAL[c] | Tyrosine ammonia-lyase | Tyr[c] -> ammonia[c] + 4Coum[c] + H+[c] | Lignin biosynthesis | 4.3.1.25 |
| CCR1[c] | cinnamoyl-CoA reductase | 4CoumCoA[c] + NADPH[c] + H+[c] -> CoumAld[c] + NADP+[c] + CoA[c] | Lignin biosynthesis | 1.2.1.44 |
| 4CL[c] | 4-coumarate--CoA ligase | CoA[c] + 4Coum[c] + ATP[c] -> 4CoumCoA[c] + ppi[c] + AMP[c] | Lignin biosynthesis | 6.2.1.12 |
| CAMT[c] | Caffeoyl-CoA O-methyltransferase | Caff-CoA[c] + S-Ade-L-meth[c] -> S-Ade-L-H[c] + Fer-CoA[c] | Lignin biosynthesis | 2.1.1.104 |

| | | | | |
|---|---|---|---|---|
| CCQT[c] | Caffeoyl-CoA:quinate O-(3,4-dihydroxycinnamoyl)transferase | CaffQuin[c] + CoA[c] -> Caff-CoA[c] + Quinate[c] | Lignin biosynthesis | 3.1.2.- |
| FCR[c] | feruloyl-CoA reductase | Fer-CoA[c] + NADPH[c] + H+[c] -> Conald[c] + NADP+[c] + CoA[c] | Lignin biosynthesis | 1.2.1.44 |
| C4H[c] | Trans-cinnamate 4-monooxygenase | t-Cinn[c] + O2[c] + NADPH[c] + H+[c] -> H2O[c] + NADP+[c] + 4Coum[c] | Lignin biosynthesis | 1.14.13.11 |
| HCT[c] | Shikimate O-hydroxycinnamoyltransferase | 4CoumCoA[c] + Shikimate[c] -> 4CoumShiki[c] + CoA[c] | Lignin biosynthesis | 2.3.1.133 |
| CAD1[c] | cinnamyl-alcohol dehydrogenase | Conald[c] + NADPH[c] + H+[c] -> Conalc[c] + NADP+[c] | Lignin biosynthesis | 1.1.1.195 |
| CAD2[c] | cinnamyl-alcohol dehydrogenase | SinaAld[c] + NADPH[c] + H+[c] -> SinapAlc[c] + NADP+[c] | Lignin biosynthesis | 1.1.1.195 |
| CQMOX[c] | coumaroylquinate 3'-monooxygenase | 4CoumQuin[c] + NADPH[c] + O2[c] + H+[c] -> CaffQuin[c] + NADP+[c] + H2O[c] | Lignin biosynthesis | 1.14.13.36 |
| FH[c] | ferulate 5-hydroxylase | Conald[c] + NADPH[c] + O2[c] + H+[c] -> 5hConif[c] + NADP+[c] + H2O[c] | Lignin biosynthesis | 1.14.13.- |
| AMETH[c] | S-adenosyl-L-methionine:3,4-dihydroxy-trans-cinnamate 3-O-methyltransferase | 5hConif[c] + S-Ade-L-meth[c] -> S-Ade-L-H[c] + SinaAld[c] + H+[c] | Lignin biosynthesis | 2.1.1.68 |
| CSMOX[c] | coumaroylshikimate 3'-monooxygenase | 4CoumShiki[c] + NADPH[c] + O2[c] + H+[c] -> CaffShiki[c] + NADP+[c] + H2O[c] | Lignin biosynthesis | 1.14.13.36 |
| CCQH[c] | p-coumaroyl-CoA:quinate hydroxycinnamoyltransferase | 4CoumCoA[c] + Quinate[c] -> 4CoumQuin[c] + CoA[c] | Lignin biosynthesis | 2.3.1.133 |
| HCCST[c] | Hydroxycinnamoyl-CoA:shikimate hydroxycinnamoyltransferase | CaffShiki[c] + CoA[c] -> Caff-CoA[c] + Shikimate[c] | Lignin biosynthesis | 3.1.2.- |
| CAD3[c] | cinnamyl-alcohol dehydrogenase | CoumAld[c] + NADPH[c] + H+[c] -> Coum-Alc[c] + NADP+[c] | Lignin biosynthesis | 1.1.1.195 |
| TCM01 | THF transporter | THF[m] -> THF[c] | transport | |
| TCM02 | Formate transporter | Formate[c] <-> Formate[m] | transport | |
| TCM03 | AMP transporter | AMP[m] + ATP[c] + 2 H+[c] -> AMP[c] + ATP[m] + 2 H+[m] | transport | |
| TCM04 | Pyruvate transporter | Pyr[c] + H+[c] -> Pyr[m] + H+[m] | transport | |

| | | | | |
|---|---|---|---|---|
| TCM05 | Aspartate transporter | Asp[m] + H+[m] <-> Asp[c] + H+[c] | transport | |
| TCM06 | Dihydroorotate transporter | Dhooro[c] -> Dhooro[m] | transport | |
| TCM07 | Orotate transporter | Orot[m] -> Orot[c] | transport | |
| TCM08 | Alanine transporter | Ala[m] + H+[m] <-> Ala[c] + H+[c] | transport | |
| TCM09 | Water transporter | H2O[c] <-> H2O[m] | transport | |
| TCM10 | Carbon dioxide transporter | CO2[c] <-> CO2[m] | transport | |
| TCM11 | Ammonia transporter | ammonia[c] <-> ammonia[m] | transport | |
| TCM12 | Oxygen transporter | O2[c] <-> O2[m] | transport | |
| TCM13 | Proton/Phosphate transporter | pi[c] + H+[c] <-> pi[m] + H+[m] | transport | |
| TCM14 | Malate/Oxaloacetate transporter | Malate[m] + OAA[c] -> Malate[c] + OAA[m] | transport | |
| TCM15 | Hydrogen peroxide transporter | H2O2[c] <-> H2O2[m] | transport | |
| TCM16 | ATP/ADP transporter | ATP[m] + ADP[c] + H+[c] -> ATP[c] + ADP[m] + H+[m] | transport | |
| TCM17 | GABA/proton transporter | GABA[c] + H+[m] <-> GABA[m] + H+[c] | transport | |
| TCM18 | Succinate/Fumarate transporter | Succ[m] + Fumr[c] <-> Fumr[m] + Succ[c] | transport | |
| TCM19 | Glycine transporter | Gly[m] + H+[m] <-> Gly[c] + H+[c] | transport | |
| TCM20 | Serine transporter | Ser[m] + H+[m] <-> Ser[c] + H+[c] | transport | |
| TCM21 | Succinate/Malate transporter | Succ[c] + Malate[m] -> Succ[m] + Malate[c] | transport | |
| TCM22 | Citrate/Malate transporter | Citrate[m] + Malate[c] -> Citrate[c] + Malate[m] | transport | |
| TCM23 | Malate/phosphate transporter | Malate[c] + pi[m] -> pi[c] + Malate[m] | transport | |
| TCM24 | Malate/alpha-Ketoglutarate transporter | Malate[c] + aKG[m] -> Malate[m] + aKG[c] | transport | |
| TCM25 | CoA transporter | CoA[c] -> CoA[m] | transport | |
| TCM26 | Acetate transporter | Acetate[c] -> Acetate[m] | transport | |
| TCM27 | Aspartate/Glutamate transporter | Asp[m] + Glu[c] <-> Asp[c] + Glu[m] | transport | |

| | | | | | |
|---|---|---|---|---|---|
| TCP01 | Aspartate transporter | Asp[p] + H+[p] <-> Asp[c] + H+[c] | transport | | |
| TCP02 | Cysteine transporter | Cys[p] + H+[p] <-> Cys[c] + H+[c] | transport | | |
| TCP03 | Glycine transporter | Gly[p] + H+[p] <-> Gly[c] + H+[c] | transport | | |
| TCP04 | Histidine transporter | His[p] + H+[p] <-> His[c] + H+[c] | transport | | |
| TCP05 | Isoleucine transporter | Ile[p] + H+[p] <-> Ile[c] + H+[c] | transport | | |
| TCP06 | Leucine transporter | Leu[p] + H+[p] <-> Leu[c] + H+[c] | transport | | |
| TCP07 | Lysine transporter | Lys[p] + H+[p] <-> Lys[c] + H+[c] | transport | | |
| TCP08 | Methionine transporter | Met[p] + H+[p] <-> Met[c] + H+[c] | transport | | |
| TCP09 | Phenylalanine transporter | Phe[p] + H+[p] <-> Phe[c] + H+[c] | transport | | |
| TCP10 | Proline transporter | Pro[p] + H+[p] <-> Pro[c] + H+[c] | transport | | |
| TCP11 | Serine transporter | Ser[p] + H+[p] <-> Ser[c] + H+[c] | transport | | |
| TCP12 | Threonine transporter | Thr[p] + H+[p] <-> Thr[c] + H+[c] | transport | | |
| TCP13 | Tryptophan transporter | Trp[p] + H+[p] <-> Trp[c] + H+[c] | transport | | |
| TCP14 | Tyrosine transporter | Tyr[p] + H+[p] <-> Tyr[c] + H+[c] | transport | | |
| TCP15 | Valine transporter | Val[p] + H+[p] <-> Val[c] + H+[c] | transport | | |
| TCP16 | p-aminobenzoate transporter | p-Abenz[p] -> p-Abenz[c] | transport | | |
| TCP17 | Tetrahydrofolate transporter | THF[c] -> THF[p] | transport | | |
| TCP18 | Formate transporter | Formate[c] <-> Formate[p] | transport | | |
| TCP19 | Glucose transporter | a-Glc[p] -> a-Glc[c] | transport | | |
| TCP20 | Hexose Phosphate/Phosphate translocator (GPT) | a-Glc1P[c] + pi[p] <-> a-Glc1P[p] + pi[c] | transport | | |
| TCP21 | Hexose Phosphate/Phosphate translocator (GPT) | a-Glc6P[c] + pi[p] <-> a-Glc6P[p] + pi[c] | transport | | |
| TCP22 | Triose phosphate-3-phosphoglycerate-phosphate translocator (TPT) | DHAP[c] + pi[p] <-> DHAP[p] + pi[c] | transport | | |

| | | | | | |
|---|---|---|---|---|---|
| TCP23 | Triose phosphate-3-phosphoglycerate-phosphate translocator (TPT) | 3PG[p] + pi[c] <-> 3PG[c] + pi[p] | transport | | |
| TCP24 | Phosphoenolpyruvate/Phosphate translocator (PPT) | PEP[c] + pi[p] -> PEP[p] + pi[c] | transport | | |
| TCP25 | AMP transporter | AMP[p] + ATP[c] + 2 H+[c] -> AMP[c] + ATP[p] + 2 H+[p] | transport | | |
| TCP26 | Dihydroorotate transporter | DHooro[p] -> DHooro[c] | transport | | |
| TCP27 | Orotate transporter | Orot[c] -> Orot[p] | transport | | |
| TCP28 | UTP transporter | UTP[p] <-> UTP[c] | transport | | |
| TCP29 | Water transporter | H2O[c] <-> H2O[p] | transport | | |
| TCP30 | Carbon dioxide transporter | CO2[c] <-> CO2[p] | transport | | |
| TCP31 | Glutamate/Glutamine transporter | Gln[p] + Glu[c] -> Glu[p] + Gln[c] | transport | | |
| TCP32 | Ammonia transporter | ammonia[c] <-> ammonia[p] | transport | | |
| TCP33 | Oxygen transporter | O2[c] <-> O2[p] | transport | | |
| TCP34 | Phosphate transporter | pi[c] + H+[c] <-> pi[p] + H+[p] | transport | | |
| TCP35 | Sulfate transporter | sulfate[c] -> sulfate[p] | transport | | |
| TCP36 | Homocysteine transporter | HomoCys[p] -> HomoCys[c] | transport | | |
| TCP37 | ATP/ADP transporter | ATP[p] + ADP[c] + H+[c] <-> ATP[c] + ADP[p] + H+[p] | transport | | |
| TCP38 | Glycolate/Glycerate transporter | 2 Glycolate[p] + Glycerate[c] <-> 2 Glycolate[c] + Glycerate[p] | transport | | |
| TCP39 | Malate/Fumarate transporter | Malate[p] + Fumr[c] <-> Fumr[p] + Malate[c] | transport | | |
| TCP40 | Oxaloacetate/Malate transporter | Malate[p] + OAA[c] -> OAA[p] + Malate[c] | transport | | |
| TCP41 | alpha-Ketoglutarate/Malate transporter | Malate[p] + aKG[c] -> aKG[p] + Malate[c] | transport | | |
| TCP42 | Glutamate/Malate transporter | Malate[c] + Glu[p] -> Glu[c] + Malate[p] | transport | | |
| TCP43 | FA transporter | Palmt[p] -> Palmt[c] | transport | | |

| | | | | |
|---|---|---|---|---|
| TCP44 | FA transporter | Steat[p] -> Steat[c] | transport | |
| TCP45 | FA transporter | Olet[p] -> Olet[c] | transport | |
| TCP46 | FA transporter | Lolet[p] -> Lolet[c] | transport | |
| TCP47 | FA transporter | Lolnt[p] -> Lolnt[c] | transport | |
| TCP48 | CoA transporter | CoA[c] -> CoA[p] | transport | |

## Metabolites abbreviations

| Abbreviation | Metabolite | KEGG ID | Compartment |
|---|---|---|---|
| 1,3-DPGA[c] | 1,3-diphosphoglycerate | C00236 | Cytosol |
| 1,3-DPGA[p] | 1,3-diphosphoglycerate | C00236 | Plastid |
| 1-ocdoP[p] | 1-(o-carboxyphenylamino)-1'-deoxyribulose-5'-phosphate | C01302 | Plastid |
| 23dh3MetVal[p] | 2,3-dihydroxy-3-methylvalerate | C06007 | Plastid |
| 23dhiVal[p] | 2,3-dihydroxy-isovalerate | C04272 | Plastid |
| 2a2hButyr[p] | 2-aceto-2-hydroxy-butyrate | C06006 | Plastid |
| 2a4h6hm78dP[m] | 2-amino-4-hydroxy-6-hydroxymethyl-7,8-dihydropteridine diphosphate | C04807 | Mitochondrion |
| 2Acelac[p] | 2-acetolactate | C06010 | Plastid |
| 2iProp3Succ[p] | 2-isopropyl-3-oxosuccinate | C04236 | Plastid |
| 2iPropMal[p] | 2-isopropylmalate | C02504 | Plastid |
| 2k3MetVal[p] | 2-keto-3-methyl-valerate | C00671 | Plastid |
| 2kiCapr[p] | 2-ketoisocaproate | C00233 | Plastid |
| 2kiVal[p] | 2-keto-isovalerate | C00141 | Plastid |
| 2oxoBut[p] | 2-oxobutanoate | C00109 | Plastid |
| 2PG[c] | 2-phosphoglycerate | C00631 | Cytosol |
| 2PG[p] | 2-phosphoglycerate | C00631 | Plastid |
| 2PhosGlyco[p] | 2-phosphoglycolate | C00988 | Plastid |
| 3DHQuin[p] | 3-dehydroquinate | C00944 | Plastid |
| 3DHShiki[p] | 3-dehydro-shikimate | C02637 | Plastid |
| 3DODara7P[p] | 3-deoxy-D-arabino-heptulosonate-7-phosphate | C04691 | Plastid |
| 3HOPropCoA[c] | 3-hydroxypropionyl-CoA | C05668 | Cytosol |
| 3hprpnte[c] | 3-Hydroxypropanoate | C01013 | Cytosol |
| 3iPropMal[p] | 3-isopropylmalate | C04411 | Plastid |
| 3PG[c] | 3-phosphoglycerate | C00197 | Cytosol |
| 3PG[p] | 3-phosphoglycerate | C00197 | Plastid |
| 3Phoshpyr[p] | 3-phospho-hydroxypyruvate | C03232 | Plastid |
| 3PhosSer[p] | 3-phospho-serine | C01005 | Plastid |
| 4Coum[c] | 4-coumarate | C00811 | Cytosol |
| 4CoumCoA[c] | 4-coumaroyl-CoA | C00223 | Cytosol |
| 4CoumQuin[c] | 4-coumaroylquinate | C12208 | Cytosol |
| 4CoumShiki[c] | 4-coumaroylshikimate | C02947 | Cytosol |
| 510MeteTHF[c] | 5,10-methylene-THF | C00143 | Cytosol |
| 510MeteTHF[m] | 5,10-methylene-THF | C00143 | Mitochondrion |
| 510MeteTHF[p] | 5,10-methylene-THF | C00143 | Plastid |
| 510MetTHF[c] | 5,10-methenyl-THF | C00445 | Cytosol |
| 510MetTHF[m] | 5,10-methenyl-THF | C00445 | Mitochondrion |
| 510MetTHF[p] | 5,10-methenyl-THF | C00445 | Plastid |
| 5EnoShiki3P[p] | 5-enolpyruvyl-shikimate-3-phosphate | C01269 | Plastid |
| 5hConif[c] | 5-hydroxy-coniferaldehyde | C12204 | Cytosol |
| 5MetTHF[c] | 5-methyl-THF | C00440 | Cytosol |
| 5MetTHF[p] | 5-methyl-THF | C00440 | Plastid |

| | | | |
|---|---|---|---|
| 6-P-gluco[c] | 6-phospho-D-gluconate | C00345 | Cytosol |
| 6-P-gluco[p] | 6-phospho-D-gluconate | C00345 | Plastid |
| 78dhPte[m] | 7,8-dihydropteroate | C00921 | Mitochondrion |
| Ace-CoA[c] | acetyl-CoA | C00024 | Cytosol |
| Ace-CoA[m] | acetyl-CoA | C00024 | Mitochondrion |
| Ace-CoA[p] | acetyl-CoA | C00024 | Plastid |
| Acetald[c] | acetaldehyde | C00084 | Cytosol |
| Acetald[p] | acetaldehyde | C00084 | Plastid |
| Acetate[c] | acetate | C00033 | Cytosol |
| Acetate[m] | acetate | C00033 | Mitochondrion |
| Acetate[p] | acetate | C00033 | Plastid |
| AcrCoA[c] | Acrylyl-CoA | C00894 | Cytosol |
| Ade-5P[p] | adenosine 5'-phosphosulfate | C00224 | Plastid |
| Adenosine[c] | adenosine | C00212 | Cytosol |
| Ade-Succ[p] | adenylo-succinate | C03794 | Plastid |
| a-D-Glucur-1-P[c] | alpha-D-glucuronate 1-phosphate | C05385 | Cytosol |
| ADP[c] | ADP | C00008 | Cytosol |
| ADP[m] | ADP | C00008 | Mitochondrion |
| ADP[p] | ADP | C00008 | Plastid |
| ADP-D-Glu[p] | ADP-D-glucose | C00498 | Plastid |
| a-Glc[c] | alpha-D-glucose | C00267 | Cytosol |
| a-Glc[e] | alpha-D-glucose | C00267 | Extracellular |
| a-Glc[p] | alpha-D-glucose | C00267 | Plastid |
| a-Glc1P[c] | alpha-D-glucose 1-phosphate | C00103 | Cytosol |
| a-Glc1P[p] | alpha-D-glucose 1-phosphate | C00103 | Plastid |
| a-Glc6P[c] | alpha-D-glucose 6-phosphate | C00668 | Cytosol |
| a-Glc6P[p] | alpha-D-glucose 6-phosphate | C00668 | Plastid |
| AICAR[p] | AICAR | C04677 | Plastid |
| aKG[c] | alpha-ketoglutarate | C00026 | Cytosol |
| aKG[m] | alpha-ketoglutarate | C00026 | Mitochondrion |
| aKG[p] | alpha-ketoglutarate | C00026 | Plastid |
| Ala[c] | L-alanine | C00041 | Cytosol |
| Ala[e] | L-alanine | C00041 | Extracellular |
| Ala[m] | L-alanine | C00041 | Mitochondrion |
| ammonia[c] | ammonia | C00014 | Cytosol |
| ammonia[m] | ammonia | C00014 | Mitochondrion |
| ammonia[p] | ammonia | C00014 | Plastid |
| AMP[c] | AMP | C00020 | Cytosol |
| AMP[m] | AMP | C00020 | Mitochondrion |
| AMP[p] | AMP | C00020 | Plastid |
| Anth[p] | anthranilate | C00108 | Plastid |
| Arg[c] | L-arginine | C00062 | Cytosol |
| ArgSucc[c] | L-arginino-succinate | C03406 | Cytosol |
| Arogenate[p] | Arogenate | C00826 | Plastid |
| Asn[c] | L-asparagine | C00152 | Cytosol |

| | | | |
|---|---|---|---|
| Asn[e] | L-asparagine | C00152 | Extracellular |
| Asn[p] | L-asparagine | C00152 | Plastid |
| Asp[c] | L-aspartate | C00049 | Cytosol |
| Asp[m] | L-aspartate | C00049 | Mitochondrion |
| Asp[p] | L-aspartate | C00049 | Plastid |
| Asp4P[p] | L-aspartyl-4-phosphate | C03082 | Plastid |
| ATP[c] | ATP | C00002 | Cytosol |
| ATP[m] | ATP | C00002 | Mitochondrion |
| ATP[p] | ATP | C00002 | Plastid |
| b-Glc6P[c] | beta-D-glucose-6-phosphate | C01172 | Cytosol |
| b-Glc6P[p] | beta-D-glucose-6-phosphate | C01172 | Plastid |
| c-Aco[c] | cis-aconitate | C00417 | Cytosol |
| c-Aco[m] | cis-aconitate | C00417 | Mitochondrion |
| Caff-CoA[c] | caffeoyl-CoA | C00323 | Cytosol |
| CaffQuin[c] | caffeoylquinate | C00852 | Cytosol |
| CaffShiki[c] | caffeoylshikimate | C10434 | Cytosol |
| CarmteP[c] | carbamoyl-phosphate | C00169 | Cytosol |
| CarmteP[p] | carbamoyl-phosphate | C00169 | Plastid |
| cellulose[c] | cellulose | C00760 | Cytosol |
| Chorismate[p] | chorismate | C00251 | Plastid |
| Citrate[c] | citrate | C00158 | Cytosol |
| Citrate[m] | citrate | C00158 | Mitochondrion |
| citrulline[c] | citrulline | C00327 | Cytosol |
| CO2[c] | CO2 | C00011 | Cytosol |
| CO2[e] | CO2 | C00011 | Extracellular |
| CO2[m] | CO2 | C00011 | Mitochondrion |
| CO2[p] | CO2 | C00011 | Plastid |
| CoA[c] | coenzyme A | C00010 | Cytosol |
| CoA[m] | coenzyme A | C00010 | Mitochondrion |
| CoA[p] | coenzyme A | C00010 | Plastid |
| Conalc[c] | coniferyl alcohol | C00590 | Cytosol |
| Conald[c] | coniferyl aldehyde | C02666 | Cytosol |
| Coum-Alc[c] | coumaryl-alcohol | C02646 | Cytosol |
| CoumAld[c] | coumaraldehyde | C05608 | Cytosol |
| Cys[c] | L-cysteine | C00097 | Cytosol |
| Cys[p] | L-cysteine | C00097 | Plastid |
| cysttn[p] | cystathionine | C02291 | Plastid |
| Cyto-Oxi[m] | Cytochromes-C-Oxidized | none | Mitochondrion |
| Cyto-Red[m] | Cytochromes-C-Reduced | none | Mitochondrion |
| D-ery4P[c] | D-erythrose-4-phosphate | C00279 | Cytosol |
| D-ery4P[p] | D-erythrose-4-phosphate | C00279 | Plastid |
| D-eryigP[p] | D-erythro-imidazole-glycerol-phosphate | C04666 | Plastid |
| D-Glu-d-Lac6P[c] | D-glucono-δ-lactone-6-phosphate | C01236 | Cytosol |
| D-Glu-d-Lac6P[p] | D-glucono-δ-lactone-6-phosphate | C01236 | Plastid |
| DHAP[c] | dihydroxy-acetone-phosphate | C00111 | Cytosol |

| | | | |
|---|---|---|---|
| DHAP[p] | dihydroxy-acetone-phosphate | C00111 | Plastid |
| Dhooro[c] | dihydroorotate | C00337 | Cytosol |
| DHooro[c] | dihydroorotate | C00337 | Cytosol |
| Dhooro[m] | dihydroorotate | C00337 | Mitochondrion |
| DHooro[m] | dihydroorotate | C00337 | Mitochondrion |
| DHooro[p] | dihydroorotate | C00337 | Plastid |
| D-myo3mP[c] | D-myo-inositol (3)-monophosphate | C04006 | Cytosol |
| D-Sed-17bP[p] | D-sedoheptulose-1,7-bisphosphate | C00447 | Plastid |
| D-Sed-7P[c] | D-sedoheptulose-7-phosphate | C00281 | Cytosol |
| D-Sed-7P[p] | D-sedoheptulose-7-phosphate | C00281 | Plastid |
| Ethanol[c] | ethanol | C00469 | Cytosol |
| Ethanol[e] | ethanol | C00469 | Extracellular |
| Fer-CoA[c] | feruloyl-CoA | C00406 | Cytosol |
| Formate[c] | formate | C00058 | Cytosol |
| Formate[m] | formate | C00058 | Mitochondrion |
| Formate[p] | formate | C00058 | Plastid |
| Fruct[c] | fructose | C00095 | Cytosol |
| Fruct16bP[c] | fructose-1,6-bisphosphate | C00354 | Cytosol |
| Fruct16bP[p] | fructose-1,6-bisphosphate | C00354 | Plastid |
| Fruct26bP[c] | fructose-2,6-bisphosphate | C00665 | Cytosol |
| Fruct26bP[p] | fructose-2,6-bisphosphate | C00665 | Plastid |
| Fruct6P[c] | fructose-6-phosphate | C00085 | Cytosol |
| Fruct6P[p] | fructose-6-phosphate | C00085 | Plastid |
| Fumr[c] | fumarate | C00122 | Cytosol |
| Fumr[m] | fumarate | C00122 | Mitochondrion |
| Fumr[p] | fumarate | C00122 | Plastid |
| G3P[c] | D-glyceraldehyde-3-phosphate | C00661 | Cytosol |
| G3P[p] | D-glyceraldehyde-3-phosphate | C00661 | Plastid |
| GABA[c] | 4-aminobutyrate | C00334 | Cytosol |
| GABA[m] | 4-aminobutyrate | C00334 | Mitochondrion |
| GDP[c] | GDP | C00035 | Cytosol |
| GDP[p] | GDP | C00035 | Plastid |
| Gln[c] | L-glutamine | C00064 | Cytosol |
| Gln[e] | L-glutamine | C00064 | Extracellular |
| Gln[p] | L-glutamine | C00064 | Plastid |
| Glu[c] | L-glutamate | C00025 | Cytosol |
| Glu[m] | L-glutamate | C00025 | Mitochondrion |
| Glu[p] | L-glutamate | C00025 | Plastid |
| glucan[p] | Large-branched-glucans | none | Plastid |
| Glucuronate[c] | glucuronate | C00191 | Cytosol |
| GludiS[p] | glutathione disulfide | C00127 | Plastid |
| Gluta[p] | glutathione | C00051 | Plastid |
| Gly[c] | glycine | C00037 | Cytosol |
| Gly[m] | glycine | C00037 | Mitochondrion |
| Gly[p] | glycine | C00037 | Plastid |

| | | | |
|---|---|---|---|
| Glycerate[c] | glycerate | C00258 | Cytosol |
| Glycerate[p] | glycerate | C00258 | Plastid |
| Glycoald[c] | glycolaldehyde | C00266 | Cytosol |
| Glycolate[c] | glycolate | C00160 | Cytosol |
| Glycolate[p] | glycolate | C00160 | Plastid |
| Glyoxylate[c] | glyoxylate | C00048 | Cytosol |
| Glyoxylate[p] | glyoxylate | C00048 | Plastid |
| GTP[c] | GTP | C00044 | Cytosol |
| GTP[p] | GTP | C00044 | Plastid |
| H+[c] | H+ | C00080 | Cytosol |
| H+[e] | H+ | C00080 | Extracellular |
| H+[m] | H+ | C00080 | Mitochondrion |
| H+[p] | H+ | C00080 | Plastid |
| H2O[c] | H2O | C00001 | Cytosol |
| H2O[e] | H2O | C00001 | Extracellular |
| H2O[m] | H2O | C00001 | Mitochondrion |
| H2O[p] | H2O | C00001 | Plastid |
| H2O2[c] | H2O2 | C00027 | Cytosol |
| H2O2[m] | H2O2 | C00027 | Mitochondrion |
| H2O2[p] | H2O2 | C00027 | Plastid |
| HCO3[c] | Bicarbonate | C00288 | Cytosol |
| HCO3[p] | Bicarbonate | C00288 | Plastid |
| His[c] | L-histidine | C00388 | Cytosol |
| His[p] | L-histidine | C00388 | Plastid |
| His-al[p] | histidinal | C01929 | Plastid |
| His-ol[p] | histidinol | C00860 | Plastid |
| HomoCys[c] | L-homocysteine | C00155 | Cytosol |
| HomoCys[p] | L-homocysteine | C00155 | Plastid |
| HomoSer[p] | homoserine | C00263 | Plastid |
| Hydpyr[c] | hydroxypyruvate | C00168 | Cytosol |
| iaP[p] | imidazole acetol-phosphate | C01267 | Plastid |
| Ile[c] | L-isoleucine | C00407 | Cytosol |
| Ile[p] | L-isoleucine | C00407 | Plastid |
| IMP[p] | inosine-5'-phosphate | C00130 | Plastid |
| ind3Ace-GP[p] | indole-3-glycerol-phosphate | C03506 | Plastid |
| Indole[p] | indole | C00463 | Plastid |
| Isocit[c] | isocitrate | C00451 | Cytosol |
| Isocit[m] | isocitrate | C00451 | Mitochondrion |
| L-23-DHDC[p] | L-2,3-dihydrodipicolinate | C03340 | Plastid |
| Lactate[c] | L-lactate | C00186 | Cytosol |
| Lactate[e] | L-lactate | C00186 | Extracellular |
| L-Asp-sAld[p] | L-aspartate-semialdehyde | C00441 | Plastid |
| Leu[c] | L-leucine | C00123 | Cytosol |
| Leu[p] | L-leucine | C00123 | Plastid |
| L-Glut-5P[c] | L-glutamate-5-phosphate | C03287 | Cytosol |

| | | | |
|---|---|---|---|
| L-Glut-5P[p] | L-glutamate-5-phosphate | C03287 | Plastid |
| L-Glut-gsAld[c] | L-glutamate-gamma-semialdehyde | C01165 | Cytosol |
| L-Glut-gsAld[p] | L-glutamate-gamma-semialdehyde | C01165 | Plastid |
| L-HisP[p] | L-histidinol-phosphate | C01100 | Plastid |
| LLdapime[p] | L,L-diaminopimelate | C00666 | Plastid |
| Lole-CoA[c] | Linoleic CoA | C02050 | Cytosol |
| Lolet[c] | Linoleic acid | C01595 | Cytosol |
| Lolet[p] | Linoleic acid | C01595 | Plastid |
| Loln-CoA[c] | Linolenic CoA | C16162 | Cytosol |
| Lolnt[c] | Linolenic acid | C06427 | Cytosol |
| Lolnt[p] | Linolenic acid | C06427 | Plastid |
| Lys[c] | L-lysine | C00047 | Cytosol |
| Lys[p] | L-lysine | C00047 | Plastid |
| Malate[c] | malate | C00149 | Cytosol |
| Malate[m] | malate | C00149 | Mitochondrion |
| Malate[p] | malate | C00149 | Plastid |
| Mal-CoA[p] | Malonyl-CoA | C00083 | Plastid |
| Mal-sAld[c] | Malonate semialdehyde | C00222 | Cytosol |
| Met[c] | L-methionine | C00073 | Cytosol |
| Met[p] | L-methionine | C00073 | Plastid |
| m-Ino[c] | myo-inositol | C00137 | Cytosol |
| mPime[p] | meso-diaminopimelate | C00680 | Plastid |
| N10FormTHF[c] | N10-formyl-THF | C00234 | Cytosol |
| N10FormTHF[m] | N10-formyl-THF | C00234 | Mitochondrion |
| N10FormTHF[p] | N10-formyl-THF | C00234 | Plastid |
| N5PAnth[p] | N-(5'-phosphoribosyl)-anthranilate | C04302 | Plastid |
| NAceGlutP[c] | N-acetylglutamyl-phosphate | C04133 | Cytosol |
| N-Ace-L-glutt[c] | N-acetyl-L-glutamate | C00624 | Cytosol |
| NAce-L-Orn[c] | N-acetyl-L-ornithine | C00437 | Cytosol |
| NAD+[c] | NAD+ | C00003 | Cytosol |
| NAD+[m] | NAD+ | C00003 | Mitochondrion |
| NAD+[p] | NAD+ | C00003 | Plastid |
| NADH[c] | NADH | C00004 | Cytosol |
| NADH[m] | NADH | C00004 | Mitochondrion |
| NADH[p] | NADH | C00004 | Plastid |
| NADP+[c] | NADP+ | C00006 | Cytosol |
| NADP+[m] | NADP+ | C00006 | Mitochondrion |
| NADP+[p] | NADP+ | C00006 | Plastid |
| NADPH[c] | NADPH | C00005 | Cytosol |
| NADPH[m] | NADPH | C00005 | Mitochondrion |
| NADPH[p] | NADPH | C00005 | Plastid |
| N-A-L-gluttsemald[c] | N-acetyl-L-glutamate 5-semialdehyde | C01250 | Cytosol |
| N-CarbL-Asp[p] | N-carbamoyl-L-aspartate | C00438 | Plastid |
| O2[c] | O2 | C00007 | Cytosol |
| O2[e] | O2 | C00007 | Extracellular |

| | | | |
|---|---|---|---|
| O2[m] | O2 | C00007 | Mitochondrion |
| O2[p] | O2 | C00007 | Plastid |
| O5P[p] | orotidine-5'-phosphate | C01103 | Plastid |
| OAA[c] | oxaloacetate | C00036 | Cytosol |
| OAA[m] | oxaloacetate | C00036 | Mitochondrion |
| OAA[p] | oxaloacetate | C00036 | Plastid |
| O-Ace-L-ser[p] | O-acetyl-L-serine | C00979 | Plastid |
| Ole-CoA[c] | Oleic CoA | C00510 | Cytosol |
| Olet[c] | Oleic acid | C00712 | Cytosol |
| Olet[p] | Oleic acid | C00712 | Plastid |
| O-P-L-hmser[p] | O-phospho-L-homoserine | C01102 | Plastid |
| Orn[c] | L-ornithine | C00077 | Cytosol |
| Orot[c] | orotate | C00295 | Cytosol |
| Orot[m] | orotate | C00295 | Mitochondrion |
| Orot[p] | orotate | C00295 | Plastid |
| oxiferr[p] | Oxidized-ferredoxins | none | Plastid |
| p-Abenz[c] | p-aminobenzoate | C00568 | Cytosol |
| p-Abenz[m] | p-aminobenzoate | C00568 | Mitochondrion |
| p-Abenz[p] | p-aminobenzoate | C00568 | Plastid |
| Palm-CoA[c] | Palmityl-CoA | C00154 | Cytosol |
| Palmt[c] | Palmitic acid | C00249 | Cytosol |
| Palmt[p] | Palmitic acid | C00249 | Plastid |
| PEP[c] | phosphoenolpyruvate | C00074 | Cytosol |
| PEP[p] | phosphoenolpyruvate | C00074 | Plastid |
| Phe[c] | L-phenylalanine | C00079 | Cytosol |
| Phe[p] | L-phenylalanine | C00079 | Plastid |
| photon[e] | photon | none | Extracellular |
| photon[p] | photon | none | Plastid |
| pi[c] | Orthophosphate | C00009 | Cytosol |
| pi[e] | Orthophosphate | C00009 | Extracellular |
| pi[m] | Orthophosphate | C00009 | Mitochondrion |
| pi[p] | Orthophosphate | C00009 | Plastid |
| ppi[c] | Pyrophosphate | C00013 | Cytosol |
| ppi[m] | Pyrophosphate | C00013 | Mitochondrion |
| ppi[p] | Pyrophosphate | C00013 | Plastid |
| Pr-AMP[p] | phosphoribosyl-AMP | C02741 | Plastid |
| Pr-ATP[p] | phosphoribosyl-ATP | C02739 | Plastid |
| Pr-BulSylFormP[p] | phosphoribulosylformimino-AICAR-P | C04916 | Plastid |
| Prep[p] | prephenate | C00254 | Plastid |
| Pr-FormCarb[p] | phosphoribosyl-formamido-carboxamide | C04734 | Plastid |
| Pr-FormCarbP[p] | phosphoribosylformiminoAICAR-phosphate | C04896 | Plastid |
| Pro[c] | L-proline | C00148 | Cytosol |
| Pro[p] | L-proline | C00148 | Plastid |
| PropCoA[c] | Propanoyl-CoA | C00100 | Cytosol |
| PRpi[p] | 5-Phospho-alpha-D-ribose | C00119 | Plastid |

| | | | |
|---|---|---|---|
| Pyr[c] | pyruvate | C00022 | Cytosol |
| Pyr[m] | pyruvate | C00022 | Mitochondrion |
| Pyr[p] | pyruvate | C00022 | Plastid |
| pyrdeh1[m] | Pyruvate-dehydrogenase-lipoate | C15972 | Mitochondrion |
| pyrdeh1[p] | Pyruvate-dehydrogenase-lipoate | C15972 | Plastid |
| pyrdeh2[m] | Pyruvate-dehydrogenase-acetylDHlipoyl | C16255 | Mitochondrion |
| pyrdeh2[p] | Pyruvate-dehydrogenase-acetylDHlipoyl | C16255 | Plastid |
| pyrdeh3[m] | Pyruvate-dehydrogenase-dihydrolipoate | C15973 | Mitochondrion |
| pyrdeh3[p] | Pyruvate-dehydrogenase-dihydrolipoate | C15973 | Plastid |
| Pyrr5Carb[c] | pyrroline5-carboxylate | C03912 | Cytosol |
| Pyrr5Carb[p] | pyrroline5-carboxylate | C03912 | Plastid |
| Q[m] | Ubiquinones | C00399 | Mitochondrion |
| QH2[m] | Ubiquinols | C00390 | Mitochondrion |
| Quinate[c] | quinate | C00296 | Cytosol |
| R5P[c] | D-ribose-5-phosphate | C00117 | Cytosol |
| R5P[p] | D-ribose-5-phosphate | C00117 | Plastid |
| redferr[p] | Reduced-ferredoxins | none | Plastid |
| Ru5P[c] | D-ribulose-5-phosphate | C00199 | Cytosol |
| Ru5P[p] | D-ribulose-5-phosphate | C00199 | Plastid |
| RuBP[p] | D-ribulose-1,5-bisphosphate | C01182 | Plastid |
| S-Ade-L-H[c] | S-adenosyl-L-homocysteine | C00021 | Cytosol |
| S-Ade-L-meth[c] | S-adenosyl-L-methionine | C00019 | Cytosol |
| Ser[c] | L-serine | C00065 | Cytosol |
| Ser[m] | L-serine | C00065 | Mitochondrion |
| Ser[p] | L-serine | C00065 | Plastid |
| SGly3P[c] | Glycerol-3-phosphate | C00093 | Cytosol |
| Shikimate[c] | shikimate | C00493 | Cytosol |
| Shikimate[p] | shikimate | C00493 | Plastid |
| Shikm3P[p] | shikimate-3-phosphate | C03175 | Plastid |
| SinaAld[c] | sinapaldehyde | C05610 | Cytosol |
| SinapAlc[c] | sinapyl-alcohol | C02325 | Cytosol |
| SMLM[c] | S-methyl-L-methionine | C03172 | Cytosol |
| Starch[e] | Starch | C00369 | Extracellular |
| Starch[p] | Starch | C00369 | Plastid |
| Stea-CoA[c] | stearoyl-CoA | C00412 | Cytosol |
| Steat[c] | Stearic acid | C01530 | Cytosol |
| Steat[p] | Stearic acid | C01530 | Plastid |
| Succ[c] | succinate | C00042 | Cytosol |
| Succ[e] | succinate | C00042 | Extracellular |
| Succ[m] | succinate | C00042 | Mitochondrion |
| Suc-CoA[m] | succinyl-CoA | C00091 | Mitochondrion |
| SuccsAld[m] | succinate semialdehyde | C00232 | Mitochondrion |
| Sucr6P[c] | sucrose-6-phosphate | C02591 | Cytosol |
| Sucrose[c] | sucrose | C00089 | Cytosol |
| Sucrose[e] | sucrose | C00089 | Extracellular |

| | | | |
|---|---|---|---|
| sulfate[c] | sulfate | C00059 | Cytosol |
| sulfate[e] | sulfate | C00059 | Extracellular |
| sulfate[p] | sulfate | C00059 | Plastid |
| sulfide[p] | hydrogen sulfide | C00283 | Plastid |
| sulfite[p] | sulfite | C00094 | Plastid |
| TAG[c] | Triacylglycerides | none | Cytosol |
| t-Cinn[c] | trans-cinnamate | C00423 | Cytosol |
| Thdpico[p] | tetrahydrodipicolinate | C03972 | Plastid |
| THF[c] | tetrahydrofolate | C00101 | Cytosol |
| THF[m] | tetrahydrofolate | C00101 | Mitochondrion |
| THF[p] | tetrahydrofolate | C00101 | Plastid |
| Thr[c] | L-threonine | C00188 | Cytosol |
| Thr[p] | L-threonine | C00188 | Plastid |
| Trp[c] | L-tryptophan | C00078 | Cytosol |
| Trp[p] | L-tryptophan | C00078 | Plastid |
| Tyr[c] | L-tyrosine | C00082 | Cytosol |
| Tyr[p] | L-tyrosine | C00082 | Plastid |
| UDP[c] | UDP | C00015 | Cytosol |
| UDP[p] | UDP | C00015 | Plastid |
| UDP-Gal[c] | UDP-galactose | C00052 | Cytosol |
| UDP-Glc[c] | UDP-D-glucose | C00029 | Cytosol |
| UDP-Glucur[c] | UDP-D-glucuronate | C00167 | Cytosol |
| UDP-L-arab[c] | UDP-L-arabinose | C00935 | Cytosol |
| UDP-Xyl[c] | UDP-D-xylose | C00190 | Cytosol |
| UMP[p] | UMP | C00105 | Plastid |
| UTP[c] | UTP | C00075 | Cytosol |
| UTP[p] | UTP | C00075 | Plastid |
| Val[c] | L-valine | C00183 | Cytosol |
| Val[p] | L-valine | C00183 | Plastid |
| Xu5P[c] | D-xylulose-5-phosphate | C00231 | Cytosol |
| Xu5P[p] | D-xylulose-5-phosphate | C00231 | Plastid |
