# Supplementary material for: Identifying essential genes/reactions of the rice photorespiration by in silico model-based analysis
Source: Rice (N Y). 2013 Aug 13;6:20. doi: 10.1186/1939-8433-6-20 (PMC4883722; doi:10.1186/1939-8433-6-20)
Supplement: Supplementary file 2 — Additional file 2: List of essential inter-compartmental transporters and list of synthetic lethal gene pairs of rice central metabolism under normal and stressed photorespiration. (PDF 128 KB) [file 12284_2013_55_MOESM2_ESM.pdf]

Additional file 2

Identifying essential genes of the rice photorespiration by *in silico* model-based analysis

Meiyappan Lakshmanan, Bijayalaxmi Mohanty, Dong-Yup Lee

List of essential transporters

Ambient conditions	Stressed conditions
mitochondrial succinate/fumarate transporter	mitochondrial succinate/fumarate transporter
mitochondrial glycine transporter	mitochondrial glycine transporter
plastidic aspartate transporter	mitochondrial serine transporter
plastidic cysteine transporter	plastidic aspartate transporter
plastidic histidine transporter	plastidic cysteine transporter
plastidic isoleucine transporter	plastidic histidine transporter
plastidic leucine transporter	plastidic isoleucine transporter
plastidic lysine transporter	plastidic leucine transporter
plastidic phenylalanine transporter	plastidic lysine transporter
plastidic threonine transporter	plastidic phenylalanine transporter
plastidic tryptophan transporter	plastidic threonine transporter
plastidic tyrosine transporter	plastidic tryptophan transporter
plastidic valine transporter	plastidic tyrosine transporter
plastidic homocysteine transporter	plastidic valine transporter
plastidic glycolate/glycerate transporter	Homocysteine transporter
plastidic malate/fumarate transporter	plastidic homocysteine transporter
	plastidic glycolate/glycerate transporter
	plastidic malate/fumarate transporter
	plastidic glutamate/Malate transporter

List of synthetic lethal gene pairs

Ambient conditions				Stressed conditions			
ALD[c]	4.1.2.13	EPI[c]	5.1.3.15	ALD[c]	4.1.2.13	EPI[c]	5.1.3.15
ALD[c]	4.1.2.13	HXK[c]	2.7.1.1	ALD[c]	4.1.2.13	HXK[c]	2.7.1.1
ALD[c]	4.1.2.13	PGI[c]	5.3.1.9	ALD[c]	4.1.2.13	PGI[c]	5.3.1.9
ALD[c]	4.1.2.13	PGI[p]	5.3.1.9	ALD[c]	4.1.2.13	PGI[p]	5.3.1.9
ATPS[m]	3.6.3.14	ACO1[m]	4.2.1.3	ATPS[m]	3.6.3.14	ACO1[m]	4.2.1.3
ATPS[m]	3.6.3.14	ACO2[m]	4.2.1.3	ATPS[m]	3.6.3.14	ACO2[m]	4.2.1.3
ATPS[m]	3.6.3.14	AKGDH[m]	1.2.7.3	ATPS[m]	3.6.3.14	AKGDH[m]	1.2.7.3
ATPS[m]	3.6.3.14	ALD[c]	4.1.2.13	ATPS[m]	3.6.3.14	ALD[c]	4.1.2.13
ATPS[m]	3.6.3.14	ALD[p]	4.1.2.13	ATPS[m]	3.6.3.14	ALD[p]	4.1.2.13
ATPS[m]	3.6.3.14	ATPS[m]	3.6.3.14	ATPS[m]	3.6.3.14	ATPS[m]	3.6.3.14
ATPS[m]	3.6.3.14	CIN[c]	3.2.1.26	ATPS[m]	3.6.3.14	CIN[c]	3.2.1.26
ATPS[m]	3.6.3.14	CSY[m]	2.3.3.1	ATPS[m]	3.6.3.14	CSY[m]	2.3.3.1
ATPS[m]	3.6.3.14	DHPS[m]	2.5.1.15	ATPS[m]	3.6.3.14	DHPS[m]	2.5.1.15
ATPS[m]	3.6.3.14	ENO1[c]	4.2.1.11	ATPS[m]	3.6.3.14	ENO1[c]	4.2.1.11
ATPS[m]	3.6.3.14	ENO1[p]	4.2.1.11	ATPS[m]	3.6.3.14	ENO1[p]	4.2.1.11
ATPS[m]	3.6.3.14	EPI[c]	5.1.3.15	ATPS[m]	3.6.3.14	EPI[c]	5.1.3.15
ATPS[m]	3.6.3.14	FBP1[c]	3.1.3.11	ATPS[m]	3.6.3.14	FBP1[c]	3.1.3.11
ATPS[m]	3.6.3.14	FBP1[p]	3.1.3.11	ATPS[m]	3.6.3.14	FBP1[p]	3.1.3.11
ATPS[m]	3.6.3.14	FDH[c]	1.2.1.2	ATPS[m]	3.6.3.14	FDH[c]	1.2.1.2
ATPS[m]	3.6.3.14	FK[c]	2.7.1.4	ATPS[m]	3.6.3.14	FK[c]	2.7.1.4
ATPS[m]	3.6.3.14	FRDR[p]	1.18.1.3	ATPS[m]	3.6.3.14	FRDR[p]	1.18.1.3
ATPS[m]	3.6.3.14	FTHFL[c]	6.3.4.3	ATPS[m]	3.6.3.14	FTHFL[c]	6.3.4.3
ATPS[m]	3.6.3.14	FTHFL[m]	6.3.4.3	ATPS[m]	3.6.3.14	FTHFL[m]	6.3.4.3
ATPS[m]	3.6.3.14	G6PGH1[c]	1.1.1.44	ATPS[m]	3.6.3.14	G6PGH1[c]	1.1.1.44
ATPS[m]	3.6.3.14	G6PGH2[p]	1.1.1.44	ATPS[m]	3.6.3.14	G6PGH2[p]	1.1.1.44
ATPS[m]	3.6.3.14	GABA-TK[m]	2.6.1.19	ATPS[m]	3.6.3.14	GABA-TK[m]	2.6.1.19
ATPS[m]	3.6.3.14	GABA-TP[m]	2.6.1.19	ATPS[m]	3.6.3.14	GABA-TP[m]	2.6.1.19
ATPS[m]	3.6.3.14	GAD[c]	4.1.1.15	ATPS[m]	3.6.3.14	GAD[c]	4.1.1.15
ATPS[m]	3.6.3.14	GADH[c]	1.2.1.21	ATPS[m]	3.6.3.14	GADH[c]	1.2.1.21
ATPS[m]	3.6.3.14	GAPDH[c]	1.2.1.12	ATPS[m]	3.6.3.14	GAPDH[c]	1.2.1.12
ATPS[m]	3.6.3.14	GAPDH[p]	1.2.1.12	ATPS[m]	3.6.3.14	GAPDH[p]	1.2.1.12
ATPS[m]	3.6.3.14	GAPN[c]	1.2.1.9	ATPS[m]	3.6.3.14	GAPN[c]	1.2.1.9
ATPS[m]	3.6.3.14	GDH1[m]	1.4.1.4	ATPS[m]	3.6.3.14	GDH1[m]	1.4.1.4
ATPS[m]	3.6.3.14	GDH2[m]	1.4.1.2	ATPS[m]	3.6.3.14	GDH2[m]	1.4.1.2
ATPS[m]	3.6.3.14	GLN1[c]	6.3.1.2	ATPS[m]	3.6.3.14	GLN1[c]	6.3.1.2
ATPS[m]	3.6.3.14	GPX[p]	1.11.1.9	ATPS[m]	3.6.3.14	GPX[p]	1.11.1.9
ATPS[m]	3.6.3.14	HXK[c]	2.7.1.1	ATPS[m]	3.6.3.14	HXK[c]	2.7.1.1
ATPS[m]	3.6.3.14	IDH[m]	1.1.1.41	ATPS[m]	3.6.3.14	IDH[m]	1.1.1.41
ATPS[m]	3.6.3.14	IDP[c]	1.1.1.42	ATPS[m]	3.6.3.14	IDP[c]	1.1.1.42

ATPS[m]	3.6.3.14	IDP[m]	1.1.1.42	ATPS[m]	3.6.3.14	IDP[m]	1.1.1.42
ATPS[m]	3.6.3.14	IPP[c]	3.6.1.1	ATPS[m]	3.6.3.14	IPP[c]	3.6.1.1
ATPS[m]	3.6.3.14	IPP[m]	3.6.1.1	ATPS[m]	3.6.3.14	IPP[m]	3.6.1.1
ATPS[m]	3.6.3.14	IPP[p]	3.6.1.1	ATPS[m]	3.6.3.14	IPP[p]	3.6.1.1
ATPS[m]	3.6.3.14	PYK[c]	2.7.1.40	ATPS[m]	3.6.3.14	PYK[c]	2.7.1.40
ATPS[m]	3.6.3.14	PYK[p]	2.7.1.40	ATPS[m]	3.6.3.14	PYK[p]	2.7.1.40
ATPS[m]	3.6.3.14	MDH[c]	1.1.1.37	ATPS[m]	3.6.3.14	MDH[c]	1.1.1.37
ATPS[m]	3.6.3.14	MDH[m]	1.1.1.37	ATPS[m]	3.6.3.14	MDH[m]	1.1.1.37
ATPS[m]	3.6.3.14	ME6[p]	1.1.1.40	ATPS[m]	3.6.3.14	ME6[p]	1.1.1.40
ATPS[m]	3.6.3.14	MLS[p]	2.3.3.9	ATPS[m]	3.6.3.14	MLS[p]	2.3.3.9
ATPS[m]	3.6.3.14	MTHFC[m]	3.5.4.9	ATPS[m]	3.6.3.14	MTHFC[m]	3.5.4.9
ATPS[m]	3.6.3.14	MTHFD1[c]	1.5.1.15	ATPS[m]	3.6.3.14	MTHFD1[c]	1.5.1.15
ATPS[m]	3.6.3.14	MTHFD1[m]	1.5.1.15	ATPS[m]	3.6.3.14	MTHFD1[m]	1.5.1.15
ATPS[m]	3.6.3.14	MTHFD1[p]	1.5.1.15	ATPS[m]	3.6.3.14	MTHFD1[p]	1.5.1.15
ATPS[m]	3.6.3.14	MTHFD2[c]	1.5.1.5	ATPS[m]	3.6.3.14	MTHFD2[c]	1.5.1.5
ATPS[m]	3.6.3.14	MTHFD2[m]	1.5.1.5	ATPS[m]	3.6.3.14	MTHFD2[m]	1.5.1.5
ATPS[m]	3.6.3.14	MTHFD2[p]	1.5.1.5	ATPS[m]	3.6.3.14	MTHFD2[p]	1.5.1.5
ATPS[m]	3.6.3.14	PCKA[c]	4.1.1.49	ATPS[m]	3.6.3.14	PCKA[c]	4.1.1.49
ATPS[m]	3.6.3.14	PDHE[m]	2.3.1.12	ATPS[m]	3.6.3.14	PDHE[m]	2.3.1.12
ATPS[m]	3.6.3.14	PDHE[p]	2.3.1.12	ATPS[m]	3.6.3.14	PDHE[p]	2.3.1.12
ATPS[m]	3.6.3.14	PFP[c]	2.7.1.90	ATPS[m]	3.6.3.14	PFP[c]	2.7.1.90
ATPS[m]	3.6.3.14	PGI[c]	5.3.1.9	ATPS[m]	3.6.3.14	PGI[c]	5.3.1.9
ATPS[m]	3.6.3.14	PGI[p]	5.3.1.9	ATPS[m]	3.6.3.14	PGI[p]	5.3.1.9
ATPS[m]	3.6.3.14	PGK[c]	2.7.2.3	ATPS[m]	3.6.3.14	PGK[c]	2.7.2.3
ATPS[m]	3.6.3.14	PGK[p]	2.7.2.3	ATPS[m]	3.6.3.14	PGK[p]	2.7.2.3
ATPS[m]	3.6.3.14	PGLYCM[c]	5.4.2.1	ATPS[m]	3.6.3.14	PGLYCM[c]	5.4.2.1
ATPS[m]	3.6.3.14	PGLYCM[p]	5.4.2.1	ATPS[m]	3.6.3.14	PGLYCM[p]	5.4.2.1
ATPS[m]	3.6.3.14	PPC[c]	4.1.1.31	ATPS[m]	3.6.3.14	PPC[c]	4.1.1.31
ATPS[m]	3.6.3.14	PPDK1[p]	2.7.9.1	ATPS[m]	3.6.3.14	PPDK1[p]	2.7.9.1
ATPS[m]	3.6.3.14	PPDK2[c]	2.7.9.1	ATPS[m]	3.6.3.14	PPDK2[c]	2.7.9.1
ATPS[m]	3.6.3.14	PPS[c]	2.7.9.2	ATPS[m]	3.6.3.14	PPS[c]	2.7.9.2
ATPS[m]	3.6.3.14	PPS[p]	2.7.9.2	ATPS[m]	3.6.3.14	PPS[p]	2.7.9.2
ATPS[m]	3.6.3.14	PURU[c]	3.5.1.10	ATPS[m]	3.6.3.14	PURU[c]	3.5.1.10
ATPS[m]	3.6.3.14	PURU[m]	3.5.1.10	ATPS[m]	3.6.3.14	PURU[m]	3.5.1.10
ATPS[m]	3.6.3.14	PURU[p]	3.5.1.10	ATPS[m]	3.6.3.14	PURU[p]	3.5.1.10
ATPS[m]	3.6.3.14	RPE[c]	5.1.3.1	ATPS[m]	3.6.3.14	RPE[c]	5.1.3.1
ATPS[m]	3.6.3.14	RPI[c]	5.3.1.6	ATPS[m]	3.6.3.14	RPI[c]	5.3.1.6
ATPS[m]	3.6.3.14	SDH[m]	1.3.5.1	ATPS[m]	3.6.3.14	SDH[m]	1.3.5.1
ATPS[m]	3.6.3.14	SSADH1[m]	1.2.1.24	ATPS[m]	3.6.3.14	SSADH1[m]	1.2.1.24
ATPS[m]	3.6.3.14	SUCLG[m]	6.2.1.5	ATPS[m]	3.6.3.14	SUCLG[m]	6.2.1.5
ATPS[m]	3.6.3.14	SUS[c]	2.4.1.13	ATPS[m]	3.6.3.14	SUS[c]	2.4.1.13
ATPS[m]	3.6.3.14	TALA[c]	2.2.1.2	ATPS[m]	3.6.3.14	TALA[c]	2.2.1.2
ATPS[m]	3.6.3.14	TALA[p]	2.2.1.2	ATPS[m]	3.6.3.14	TALA[p]	2.2.1.2

ATPS[m]	3.6.3.14	TKT1[c]	2.2.1.1	ATPS[m]	3.6.3.14	TKT1[c]	2.2.1.1
ATPS[m]	3.6.3.14	TKT2[c]	2.2.1.1	ATPS[m]	3.6.3.14	TKT2[c]	2.2.1.1
ATPS[m]	3.6.3.14	TPI[c]	5.3.1.1	ATPS[m]	3.6.3.14	TPI[c]	5.3.1.1
ATPS[m]	3.6.3.14	TPI[p]	5.3.1.1	ATPS[m]	3.6.3.14	TPI[p]	5.3.1.1
CIN[c]	3.2.1.26	SUS[c]	2.4.1.13	CIN[c]	3.2.1.26	SUS[c]	2.4.1.13
ENO1[c]	4.2.1.11	ENO1[p]	4.2.1.11	ENO1[c]	4.2.1.11	ENO1[p]	4.2.1.11
ENO1[c]	4.2.1.11	PYK[p]	2.7.1.40	ENO1[c]	4.2.1.11	PYK[p]	2.7.1.40
ENO1[c]	4.2.1.11	MTHFC[m]	3.5.4.9	ENO1[c]	4.2.1.11	MTHFC[m]	3.5.4.9
ENO1[c]	4.2.1.11	PDHE[p]	2.3.1.12	ENO1[c]	4.2.1.11	PDHE[p]	2.3.1.12
ENO1[c]	4.2.1.11	PGLYCM[p]	5.4.2.1	ENO1[c]	4.2.1.11	PGLYCM[p]	5.4.2.1
ENO1[c]	4.2.1.11	PURU[m]	3.5.1.10	ENO1[c]	4.2.1.11	PURU[m]	3.5.1.10
ENO1[p]	4.2.1.11	PGLYCM[c]	5.4.2.1	ENO1[p]	4.2.1.11	PGLYCM[c]	5.4.2.1
EPI[c]	5.1.3.15	FBP1[c]	3.1.3.11	EPI[c]	5.1.3.15	FBP1[c]	3.1.3.11
EPI[c]	5.1.3.15	HXK[c]	2.7.1.1	EPI[c]	5.1.3.15	HXK[c]	2.7.1.1
EPI[c]	5.1.3.15	PGI[p]	5.3.1.9	EPI[c]	5.1.3.15	PGI[p]	5.3.1.9
FBP1[c]	3.1.3.11	HXK[c]	2.7.1.1	FBP1[c]	3.1.3.11	HXK[c]	2.7.1.1
FBP1[c]	3.1.3.11	PGI[c]	5.3.1.9	FBP1[c]	3.1.3.11	PGI[c]	5.3.1.9
FBP1[c]	3.1.3.11	PGI[p]	5.3.1.9	FBP1[c]	3.1.3.11	PGI[p]	5.3.1.9
FDH[c]	1.2.1.2	PYK[p]	2.7.1.40	FDH[c]	1.2.1.2	PYK[p]	2.7.1.40
FDH[c]	1.2.1.2	PDHE[p]	2.3.1.12	FDH[c]	1.2.1.2	PDHE[p]	2.3.1.12
FNR[p]	1.18.1.2	ACO1[m]	4.2.1.3	FNR[p]	1.18.1.2	ACO1[m]	4.2.1.3
FNR[p]	1.18.1.2	ACO2[m]	4.2.1.3	FNR[p]	1.18.1.2	ACO2[m]	4.2.1.3
FNR[p]	1.18.1.2	AKGDH[m]	1.2.7.3	FNR[p]	1.18.1.2	AKGDH[m]	1.2.7.3
FNR[p]	1.18.1.2	ALD[c]	4.1.2.13	FNR[p]	1.18.1.2	ALD[c]	4.1.2.13
FNR[p]	1.18.1.2	ALD[p]	4.1.2.13	FNR[p]	1.18.1.2	ALD[p]	4.1.2.13
FNR[p]	1.18.1.2	ATPS[m]	3.6.3.14	FNR[p]	1.18.1.2	ATPS[m]	3.6.3.14
FNR[p]	1.18.1.2	CIN[c]	3.2.1.26	FNR[p]	1.18.1.2	CIN[c]	3.2.1.26
FNR[p]	1.18.1.2	CSY[m]	2.3.3.1	FNR[p]	1.18.1.2	CSY[m]	2.3.3.1
FNR[p]	1.18.1.2	DHPS[m]	2.5.1.15	FNR[p]	1.18.1.2	DHPS[m]	2.5.1.15
FNR[p]	1.18.1.2	ENO1[c]	4.2.1.11	FNR[p]	1.18.1.2	ENO1[c]	4.2.1.11
FNR[p]	1.18.1.2	ENO1[p]	4.2.1.11	FNR[p]	1.18.1.2	ENO1[p]	4.2.1.11
FNR[p]	1.18.1.2	EPI[c]	5.1.3.15	FNR[p]	1.18.1.2	EPI[c]	5.1.3.15
FNR[p]	1.18.1.2	FBP1[c]	3.1.3.11	FNR[p]	1.18.1.2	FBP1[c]	3.1.3.11
FNR[p]	1.18.1.2	FBP1[p]	3.1.3.11	FNR[p]	1.18.1.2	FBP1[p]	3.1.3.11
FNR[p]	1.18.1.2	FDH[c]	1.2.1.2	FNR[p]	1.18.1.2	FDH[c]	1.2.1.2
FNR[p]	1.18.1.2	FK[c]	2.7.1.4	FNR[p]	1.18.1.2	FK[c]	2.7.1.4
FNR[p]	1.18.1.2	FNR[p]	1.18.1.2	FNR[p]	1.18.1.2	FNR[p]	1.18.1.2
FNR[p]	1.18.1.2	FRDR[p]	1.18.1.3	FNR[p]	1.18.1.2	FRDR[p]	1.18.1.3
FNR[p]	1.18.1.2	FTHFL[c]	6.3.4.3	FNR[p]	1.18.1.2	FTHFL[c]	6.3.4.3
FNR[p]	1.18.1.2	FTHFL[m]	6.3.4.3	FNR[p]	1.18.1.2	FTHFL[m]	6.3.4.3
FNR[p]	1.18.1.2	G6PGH1[c]	1.1.1.44	FNR[p]	1.18.1.2	G6PGH1[c]	1.1.1.44
FNR[p]	1.18.1.2	G6PGH2[p]	1.1.1.44	FNR[p]	1.18.1.2	G6PGH2[p]	1.1.1.44
FNR[p]	1.18.1.2	GABA-	2.6.1.19	FNR[p]	1.18.1.2	GABA-	2.6.1.19

		TK[m]				TK[m]	
FNR[p]	1.18.1.2	GABA-TP[m]	2.6.1.19	FNR[p]	1.18.1.2	GABA-TP[m]	2.6.1.19
FNR[p]	1.18.1.2	GAD[c]	4.1.1.15	FNR[p]	1.18.1.2	GAD[c]	4.1.1.15
FNR[p]	1.18.1.2	GADH[c]	1.2.1.21	FNR[p]	1.18.1.2	GADH[c]	1.2.1.21
FNR[p]	1.18.1.2	GAPDH[c]	1.2.1.12	FNR[p]	1.18.1.2	GAPDH[c]	1.2.1.12
FNR[p]	1.18.1.2	GAPDH[p]	1.2.1.12	FNR[p]	1.18.1.2	GAPDH[p]	1.2.1.12
FNR[p]	1.18.1.2	GAPN[c]	1.2.1.9	FNR[p]	1.18.1.2	GAPN[c]	1.2.1.9
FNR[p]	1.18.1.2	GDH1[m]	1.4.1.4	FNR[p]	1.18.1.2	GDH1[m]	1.4.1.4
FNR[p]	1.18.1.2	GDH2[m]	1.4.1.2	FNR[p]	1.18.1.2	GDH2[m]	1.4.1.2
FNR[p]	1.18.1.2	GLN1[c]	6.3.1.2	FNR[p]	1.18.1.2	GLN1[c]	6.3.1.2
FNR[p]	1.18.1.2	GPX[p]	1.11.1.9	FNR[p]	1.18.1.2	GPX[p]	1.11.1.9
FNR[p]	1.18.1.2	HXK[c]	2.7.1.1	FNR[p]	1.18.1.2	HXK[c]	2.7.1.1
FNR[p]	1.18.1.2	IDH[m]	1.1.1.41	FNR[p]	1.18.1.2	IDH[m]	1.1.1.41
FNR[p]	1.18.1.2	IDP[c]	1.1.1.42	FNR[p]	1.18.1.2	IDP[c]	1.1.1.42
FNR[p]	1.18.1.2	IDP[m]	1.1.1.42	FNR[p]	1.18.1.2	IDP[m]	1.1.1.42
FNR[p]	1.18.1.2	IPP[c]	3.6.1.1	FNR[p]	1.18.1.2	IPP[c]	3.6.1.1
FNR[p]	1.18.1.2	IPP[m]	3.6.1.1	FNR[p]	1.18.1.2	IPP[m]	3.6.1.1
FNR[p]	1.18.1.2	IPP[p]	3.6.1.1	FNR[p]	1.18.1.2	IPP[p]	3.6.1.1
FNR[p]	1.18.1.2	PYK[c]	2.7.1.40	FNR[p]	1.18.1.2	PYK[c]	2.7.1.40
FNR[p]	1.18.1.2	PYK[p]	2.7.1.40	FNR[p]	1.18.1.2	PYK[p]	2.7.1.40
FNR[p]	1.18.1.2	MDH[c]	1.1.1.37	FNR[p]	1.18.1.2	MDH[c]	1.1.1.37
FNR[p]	1.18.1.2	MDH[m]	1.1.1.37	FNR[p]	1.18.1.2	MDH[m]	1.1.1.37
FNR[p]	1.18.1.2	ME6[p]	1.1.1.40	FNR[p]	1.18.1.2	ME6[p]	1.1.1.40
FNR[p]	1.18.1.2	MLS[p]	2.3.3.9	FNR[p]	1.18.1.2	MLS[p]	2.3.3.9
FNR[p]	1.18.1.2	MTHFC[m]	3.5.4.9	FNR[p]	1.18.1.2	MTHFC[m]	3.5.4.9
FNR[p]	1.18.1.2	MTHFD1[c]	1.5.1.15	FNR[p]	1.18.1.2	MTHFD1[c]	1.5.1.15
FNR[p]	1.18.1.2	MTHFD1[m]	1.5.1.15	FNR[p]	1.18.1.2	MTHFD1[m]	1.5.1.15
FNR[p]	1.18.1.2	MTHFD1[p]	1.5.1.15	FNR[p]	1.18.1.2	MTHFD1[p]	1.5.1.15
FNR[p]	1.18.1.2	MTHFD2[c]	1.5.1.5	FNR[p]	1.18.1.2	MTHFD2[c]	1.5.1.5
FNR[p]	1.18.1.2	MTHFD2[m]	1.5.1.5	FNR[p]	1.18.1.2	MTHFD2[m]	1.5.1.5
FNR[p]	1.18.1.2	MTHFD2[p]	1.5.1.5	FNR[p]	1.18.1.2	MTHFD2[p]	1.5.1.5
FNR[p]	1.18.1.2	PCKA[c]	4.1.1.49	FNR[p]	1.18.1.2	PCKA[c]	4.1.1.49
FNR[p]	1.18.1.2	PDHE[m]	2.3.1.12	FNR[p]	1.18.1.2	PDHE[m]	2.3.1.12
FNR[p]	1.18.1.2	PDHE[p]	2.3.1.12	FNR[p]	1.18.1.2	PDHE[p]	2.3.1.12
FNR[p]	1.18.1.2	PFP[c]	2.7.1.90	FNR[p]	1.18.1.2	PFP[c]	2.7.1.90
FNR[p]	1.18.1.2	PGI[c]	5.3.1.9	FNR[p]	1.18.1.2	PGI[c]	5.3.1.9
FNR[p]	1.18.1.2	PGI[p]	5.3.1.9	FNR[p]	1.18.1.2	PGI[p]	5.3.1.9
FNR[p]	1.18.1.2	PGK[c]	2.7.2.3	FNR[p]	1.18.1.2	PGK[c]	2.7.2.3
FNR[p]	1.18.1.2	PGK[p]	2.7.2.3	FNR[p]	1.18.1.2	PGK[p]	2.7.2.3
FNR[p]	1.18.1.2	PGLYCM[c]	5.4.2.1	FNR[p]	1.18.1.2	PGLYCM[c]	5.4.2.1
FNR[p]	1.18.1.2	PGLYCM[p]	5.4.2.1	FNR[p]	1.18.1.2	PGLYCM[p]	5.4.2.1
FNR[p]	1.18.1.2	PPC[c]	4.1.1.31	FNR[p]	1.18.1.2	PPC[c]	4.1.1.31

FNR[p]	1.18.1.2	PPDK1[p]	2.7.9.1	FNR[p]	1.18.1.2	PPDK1[p]	2.7.9.1
FNR[p]	1.18.1.2	PPDK2[c]	2.7.9.1	FNR[p]	1.18.1.2	PPDK2[c]	2.7.9.1
FNR[p]	1.18.1.2	PPS[c]	2.7.9.2	FNR[p]	1.18.1.2	PPS[c]	2.7.9.2
FNR[p]	1.18.1.2	PPS[p]	2.7.9.2	FNR[p]	1.18.1.2	PPS[p]	2.7.9.2
FNR[p]	1.18.1.2	PURU[c]	3.5.1.10	FNR[p]	1.18.1.2	PURU[c]	3.5.1.10
FNR[p]	1.18.1.2	PURU[m]	3.5.1.10	FNR[p]	1.18.1.2	PURU[m]	3.5.1.10
FNR[p]	1.18.1.2	PURU[p]	3.5.1.10	FNR[p]	1.18.1.2	PURU[p]	3.5.1.10
FNR[p]	1.18.1.2	RPE[c]	5.1.3.1	FNR[p]	1.18.1.2	RPE[c]	5.1.3.1
FNR[p]	1.18.1.2	RPI[c]	5.3.1.6	FNR[p]	1.18.1.2	RPI[c]	5.3.1.6
FNR[p]	1.18.1.2	SDH[m]	1.3.5.1	FNR[p]	1.18.1.2	SDH[m]	1.3.5.1
FNR[p]	1.18.1.2	SSADH1[m]	1.2.1.24	FNR[p]	1.18.1.2	SSADH1[m]	1.2.1.24
FNR[p]	1.18.1.2	SUCLG[m]	6.2.1.5	FNR[p]	1.18.1.2	SUCLG[m]	6.2.1.5
FNR[p]	1.18.1.2	SUS[c]	2.4.1.13	FNR[p]	1.18.1.2	SUS[c]	2.4.1.13
FNR[p]	1.18.1.2	TALA[c]	2.2.1.2	FNR[p]	1.18.1.2	TALA[c]	2.2.1.2
FNR[p]	1.18.1.2	TALA[p]	2.2.1.2	FNR[p]	1.18.1.2	TALA[p]	2.2.1.2
FNR[p]	1.18.1.2	TKT1[c]	2.2.1.1	FNR[p]	1.18.1.2	TKT1[c]	2.2.1.1
FNR[p]	1.18.1.2	TKT2[c]	2.2.1.1	FNR[p]	1.18.1.2	TKT2[c]	2.2.1.1
FNR[p]	1.18.1.2	TPI[c]	5.3.1.1	FNR[p]	1.18.1.2	TPI[c]	5.3.1.1
FNR[p]	1.18.1.2	TPI[p]	5.3.1.1	FNR[p]	1.18.1.2	TPI[p]	5.3.1.1
FRDR[p]	1.18.1.3	GAPDH[c]	1.2.1.12	FRDR[p]	1.18.1.3	GAPDH[c]	1.2.1.12
FRDR[p]	1.18.1.3	GAPDH[p]	1.2.1.12	FRDR[p]	1.18.1.3	GAPDH[p]	1.2.1.12
FRDR[p]	1.18.1.3	MDH[c]	1.1.1.37	FRDR[p]	1.18.1.3	MDH[c]	1.1.1.37
FRDR[p]	1.18.1.3	PGK[c]	2.7.2.3	FRDR[p]	1.18.1.3	PGK[c]	2.7.2.3
FRDR[p]	1.18.1.3	PGK[p]	2.7.2.3	FRDR[p]	1.18.1.3	PGK[p]	2.7.2.3
FRDR[p]	1.18.1.3	TPI[c]	5.3.1.1	FRDR[p]	1.18.1.3	TPI[c]	5.3.1.1
FRDR[p]	1.18.1.3	TPI[p]	5.3.1.1	FRDR[p]	1.18.1.3	TPI[p]	5.3.1.1
GAPDH[c]	1.2.1.12	GAPDH[p]	1.2.1.12	GAPDH[c]	1.2.1.12	GAPDH[p]	1.2.1.12
GAPDH[c]	1.2.1.12	PGK[p]	2.7.2.3	GAPDH[c]	1.2.1.12	PGK[p]	2.7.2.3
GAPDH[c]	1.2.1.12	TPI[p]	5.3.1.1	GAPDH[c]	1.2.1.12	TPI[p]	5.3.1.1
GAPDH[p]	1.2.1.12	MDH[c]	1.1.1.37	GAPDH[p]	1.2.1.12	MDH[c]	1.1.1.37
GAPDH[p]	1.2.1.12	PGK[c]	2.7.2.3	GAPDH[p]	1.2.1.12	PGK[c]	2.7.2.3
GAPDH[p]	1.2.1.12	TPI[c]	5.3.1.1	GAPDH[p]	1.2.1.12	TPI[c]	5.3.1.1
GAPDH[p]	1.2.1.12	TPI[p]	5.3.1.1	GAPDH[p]	1.2.1.12	TPI[p]	5.3.1.1
GDH2[m]	1.4.1.2	FRDR[p]	1.18.1.3	GDH2[m]	1.4.1.2	FRDR[p]	1.18.1.3
HXK[c]	2.7.1.1	PGI[c]	5.3.1.9	GDH2[m]	1.4.1.2	TPI[c]	5.3.1.1
HXK[c]	2.7.1.1	SUS[c]	2.4.1.13	GDH2[m]	1.4.1.2	TPI[p]	5.3.1.1
IPP[c]	3.6.1.1	PPDK2[c]	2.7.9.1	HXK[c]	2.7.1.1	PGI[c]	5.3.1.9
IPP[p]	3.6.1.1	PPDK1[p]	2.7.9.1	HXK[c]	2.7.1.1	SUS[c]	2.4.1.13
IPP[p]	3.6.1.1	PPS[p]	2.7.9.2	IPP[c]	3.6.1.1	PPDK2[c]	2.7.9.1
PYK[c]	2.7.1.40	PPDK2[c]	2.7.9.1	IPP[p]	3.6.1.1	PPDK1[p]	2.7.9.1
PYK[p]	2.7.1.40	MTHFC[m]	3.5.4.9	IPP[p]	3.6.1.1	PPS[p]	2.7.9.2
PYK[p]	2.7.1.40	PGLYCM[c]	5.4.2.1	PYK[c]	2.7.1.40	PPDK2[c]	2.7.9.1
PYK[p]	2.7.1.40	PPDK1[p]	2.7.9.1	PYK[p]	2.7.1.40	MTHFC[m]	3.5.4.9

PYK[p]	2.7.1.40	PURU[m]	3.5.1.10	PYK[p]	2.7.1.40	PGLYCM[c]	5.4.2.1
MDH[c]	1.1.1.37	MDH[m]	1.1.1.37	PYK[p]	2.7.1.40	PPDK1[p]	2.7.9.1
MDH[c]	1.1.1.37	PGK[p]	2.7.2.3	PYK[p]	2.7.1.40	PURU[m]	3.5.1.10
MDH[c]	1.1.1.37	TPI[p]	5.3.1.1	MDH[c]	1.1.1.37	MDH[m]	1.1.1.37
MTHFC[m]	3.5.4.9	PDHE[p]	2.3.1.12	MDH[c]	1.1.1.37	PGK[p]	2.7.2.3
MTHFC[m]	3.5.4.9	PGLYCM[c]	5.4.2.1	MDH[c]	1.1.1.37	TPI[c]	5.3.1.1
PDHE[p]	2.3.1.12	PGLYCM[c]	5.4.2.1	MDH[c]	1.1.1.37	TPI[p]	5.3.1.1
PDHE[p]	2.3.1.12	PURU[m]	3.5.1.10	MTHFC[m]	3.5.4.9	PDHE[p]	2.3.1.12
PGI[c]	5.3.1.9	PGI[p]	5.3.1.9	MTHFC[m]	3.5.4.9	PGLYCM[c]	5.4.2.1
PGK[c]	2.7.2.3	FRDR[p]	1.18.1.3	PDHE[p]	2.3.1.12	PGLYCM[c]	5.4.2.1
PGK[c]	2.7.2.3	PGK[p]	2.7.2.3	PDHE[p]	2.3.1.12	PURU[m]	3.5.1.10
PGK[c]	2.7.2.3	TPI[p]	5.3.1.1	PGI[c]	5.3.1.9	PGI[p]	5.3.1.9
PGK[p]	2.7.2.3	TPI[c]	5.3.1.1	PGK[c]	2.7.2.3	FRDR[p]	1.18.1.3
PGK[p]	2.7.2.3	TPI[p]	5.3.1.1	PGK[c]	2.7.2.3	PGK[p]	2.7.2.3
PGLYCM[c]	5.4.2.1	PGLYCM[p]	5.4.2.1	PGK[c]	2.7.2.3	TPI[p]	5.3.1.1
PGLYCM[c]	5.4.2.1	PURU[m]	3.5.1.10	PGK[p]	2.7.2.3	TPI[c]	5.3.1.1
TPI[c]	5.3.1.1	TPI[p]	5.3.1.1	PGK[p]	2.7.2.3	TPI[p]	5.3.1.1
				PGLYCM[c]	5.4.2.1	PGLYCM[p]	5.4.2.1
				PGLYCM[c]	5.4.2.1	PURU[m]	3.5.1.10
				TPI[c]	5.3.1.1	TPI[p]	5.3.1.1
