# Supplementary figures and images for: Identifying essential genes/reactions of the rice photorespiration by in silico model-based analysis
Source: Rice (N Y). 2013 Aug 13;6:20. doi: 10.1186/1939-8433-6-20 (PMC4883722; doi:10.1186/1939-8433-6-20)

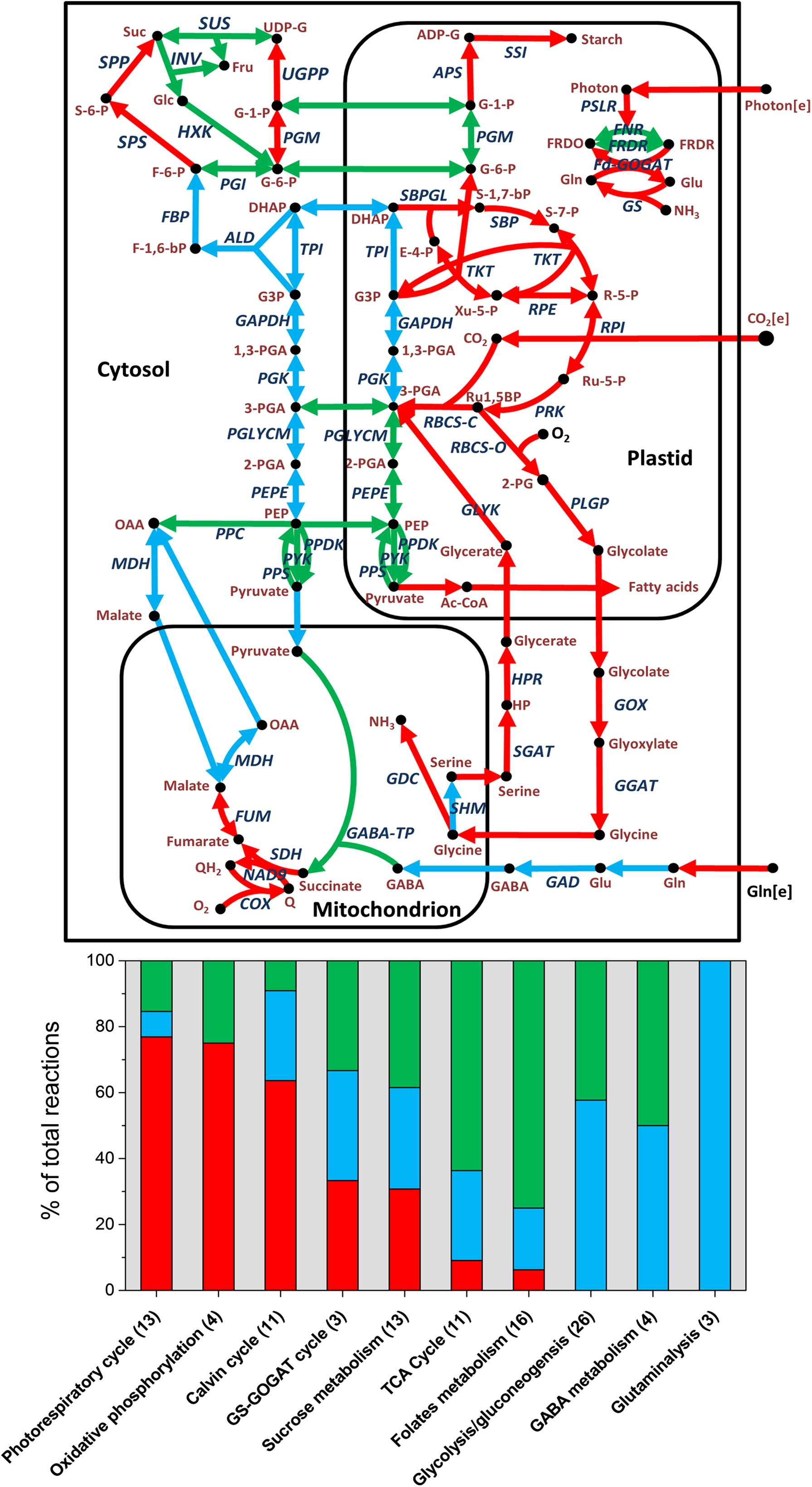

Supplement: Supplementary file 3 — Authors’ original file for figure 1 [file 12284_2013_55_MOESM3_ESM.tif]
